# The application of BMRT-HPV viral load to secondary screening strategies for cervical cancer

**DOI:** 10.1371/journal.pone.0232117

**Published:** 2020-05-01

**Authors:** Lyufang Duan, Hui Du, Chun Wang, Xia Huang, Xinfeng Qu, Bin Shi, Yan Liu, Wei Zhang, Xianzhi Duan, Lihui Wei, Jerome L. Belinson, Ruifang Wu

**Affiliations:** 1 Department of Gynecology and Obstetrics, Peking University Shenzhen Hospital, Shenzhen, China; 2 Shenzhen Key Laboratory on Technology for Early Diagnosis of Major Gynecological Diseases, Shenzhen, PR,China; 3 Sanming Project of Medicine in Shenzhen Peking University Shenzhen Hospital, Shenzhen, China; 4 Department of Gynecology and Obstetrics, The Second Hospital of Hebei Medical University, Hebei, China; 5 Department of Gynecology and Obstetrics, Huashan Hospital North, Fudan University, Shanghai, China; 6 Department of Gynecology and Obstetrics, Zhongnan Hospital of Wuhan University, Wuhan, China; 7 Department of Gynecology and Obstetrics, Capital Medical University Beijing Tongren Hospital, Beijing, PR, China; 8 Department of Gynecology and Obstetrics, Peking University People's Hospital, Beijing, PR, China; 9 Preventive Oncology International, Cleveland Heights, OH, United States of America; 10 Women’s Health Institute, Cleveland Clinic, Cleveland, OH, United States of America; Rudjer Boskovic Institute, CROATIA

## Abstract

**Objective:**

Evaluate the significance of BMRT HPV assay viral load and its performance for secondary screening.

**Methods:**

BMRT-HPV reports type-specific viral loads/10,000 cells. We tested 1,495 physician collected, stored specimens from Chinese Multiple-center Screening Trial (CHIMUST), that were positive by Cobas, SeqHPV, and/or Cytology (≥LSIL); and 2,990 age matched, negatives in a nested case control study. We explored the relationship between BMRT HR-HPV viral load and cervical lesions, determined alternative CIN2+ cut-points by ROC curve, and evaluated BMRT HR-HPV for primary / secondary cervical cancer screening.

**Results:**

The viral loads of HPV16/18, 12 other subtypes HR-HPV and 14 HR-HPV were statistically different in all grades of cervical lesions (*P*<0.05, among which HPV16, 33 and 58 showed the strongest relationship (*P*<0.01). The viral load of HR-HPV also increased with the grade of cervical lesions (*P*<0.05). The sensitivity for CIN2+ and CIN3+ of BMRT was comparable to Cobas (92.6% vs 94.3%, 100% vs 100%, *P*>0.05), specificity was higher than Cobas (84.8% vs 83.3%, 83.5% vs 82.0%, *P*<0.001). When using HPV16/18 viral load(log cut-point ≥3.2929), plus the viral-load of 12 other subtypes (log cut-point ≥3.9625) as secondary triage, compared with Cobas HPV16/18+ plus cytology ≥ASC-US as triage, the sensitivities for CIN2+ and CIN3+ were similar (*P*>0.05). However, the BMRT HR-HPV viral load combined with subtypes did not require cytology.

**Conclusion:**

BMRT is as sensitive as Cobas4800 for primary cervical cancer screening. BMRT HR-HPV viral load combined with subtypes can be used as a secondary strategy for cervical cancer screening, especially for areas with insufficient cytological resources.

## 1 Introduction

Persistent high-risk human papillomavirus(HR-HPV) is a necessary cause of cervical cancer, HPV-based cervical screening can identify >95% of pre-cancerous cervical lesions (cervical intraepithelial neoplasia [CIN] grade 2 or worse [CIN2+]) [1], so high-risk HPV testing has been widely accepted for primary screening. Compared to primary screening with cytology, a primary HR-HPV test has superior sensitivity for CIN2+, but relatively low specificity and positive predictive value (PPV). This creates a need for triage of HPV positives to minimize the false positives referred for colposcopy. Previous proposals for the triage of HR-HPV positive women include cytology, genotyping for HPV16 and HPV18, immunostaining for p16, with or without ki-67 and host or viral gene methylation [2–5]. However, these methods have their own limitations, including a relatively low sensitivity, low PPV, and subjectivity.

The relationship between HR-HPV viral load and the progression of cervical precancerous lesions remains controversial. Several studies from our team have shown that the higher the HR-HPV viral load, the higher the risk and severity of cervical lesions [6,7]. In particular, the study of Shen et. al. [7] used micro-cutting technology to accurately obtain HPV viral load in different grades of cervical lesions and confirmed that HPV viral load is a key independent indicator of high-grade disease. Recently, some authors have proposed the use of viral load alone or combined with genotyping as a triage strategy for HPV primary screening [6,8–10]. It is important to note that most of the HPV detection techniques used in these studies are semi-quantitative such as HC-II. These assays do not distinguish high-risk HPV genotypes, so it is impossible to simultaneously determine genotype and viral load.

BMRT (BioPerfectus Multiplex Real-Time PCR assay, Taizhou, China) is a PCR based assay developed in 2015. It measures the number of cervical cells by quantitatively detecting the number of single-copy genes (TOP3 genes) in cervical exfoliated cells, and then reports HPV viral load (copies/10,000 cells) for standardized quantitation. This assay can detect 14 high-risk HPV subtypes and 7 Medium and low risk subtypes; and it provides viral load per unit cell of each subtype.

In this study, we selected physician-collected specimens from the Chinese Multi-Center Screening Trial (CHIMUST) for BMRT testing. Using this assay, we investigated the relationship between viral load and cervical lesions and assessed the effectiveness of a triage strategy using HPV viral load combined with HPV subtypes.

## 2 Materials and methods

### 2.1 Study subjects

This study of BMRT-HPV was a nested case–control study within the Chinese Multi-center Cervical Cancer Screening Trial (CHIMUST) (Registration number: ChiCTR-EOC-16008456). The CHIMUST protocol was approved by the ethics committee of Peking University Shenzhen Hospital(IRB:PUSH2016001) and Cleveland Clinic Institutional Review Board (IRB:15–1549). From August 2016 to January 2018, the trial provided screening for 10885 women who had not been screened in the past 3 years. The women needed to be non-pregnant sexually active women, ages 30 to 59 years with no prior pelvic radiation, and with an intact uterus. They needed to agree to return for further testing if their screening was positive. Women were recruited from Beijing and the following five provinces: 1. Jiangxi 2. Hebei 3. Hubei 4. Guangdong 5. Inner Mongolia Autonomous Region. ALL participants signed an informed consent document before enrollment.

All participants provided a self-collected vaginal sample and a physician-collected endocervical sample. The physician-sampling was performed following self-sampling. The physician placed a vaginal speculum to expose the cervix, then obtained a cervical exfoliated cell sample at the squamocolumnar junction of the cervix with a sampling brush and then placed the brush into a 20mL PreservCyt® solution (Hologic, Marlborough Mass, USA, DOC sample) for testing. All samples were tested with the PCR-based high-risk HPV assays: Cobas and SeqHPV (BGI, Shenzhen, China). The physician-collected samples were also tested by Cytology using the Hologic I2 imager system (computer assisted cytology). Women positive by either Cobas or SeqHPV (self or direct) were referred for colposcopy.

**Fig 1:**
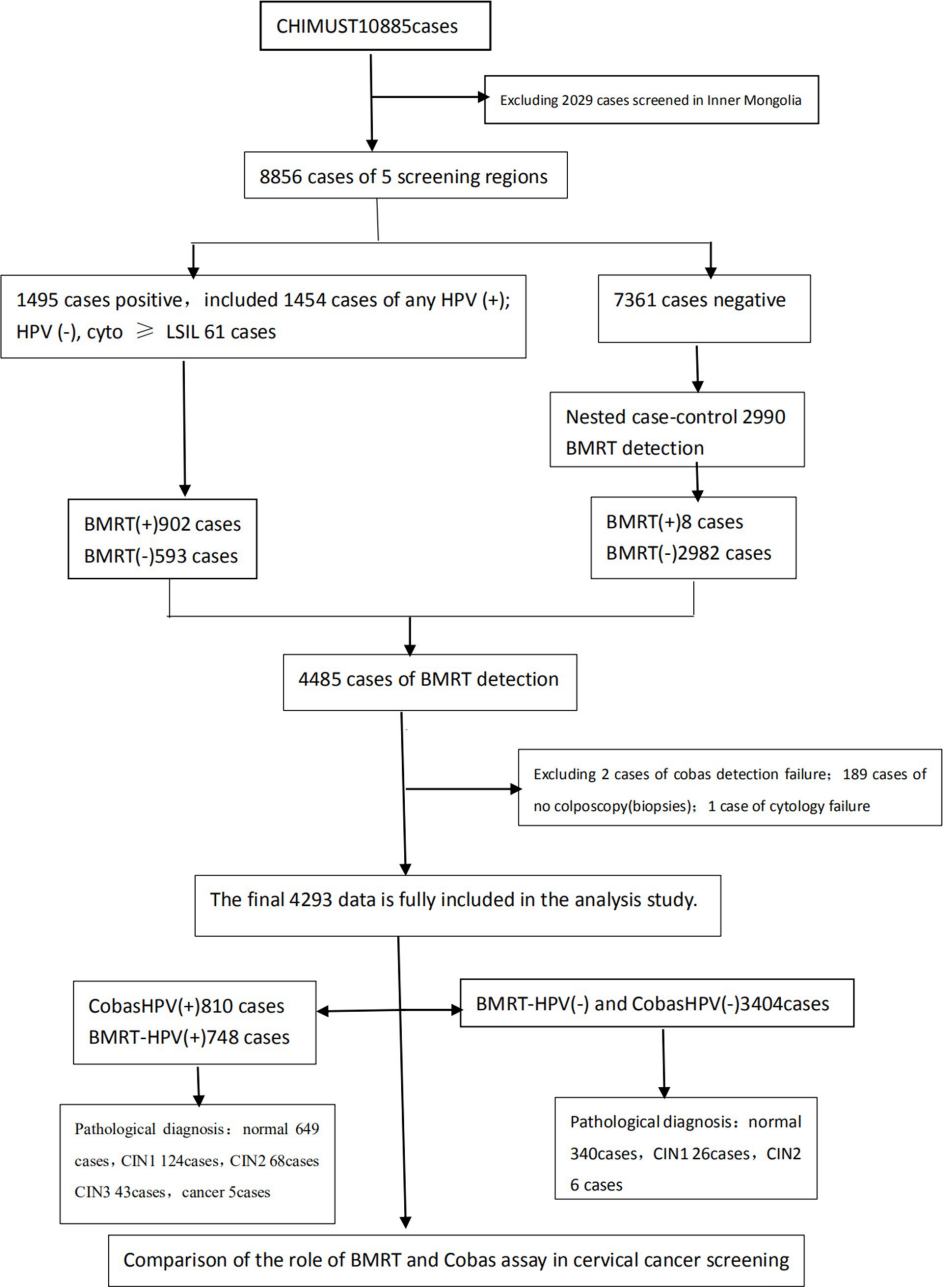
BMRT-HPV nested case–control study population.

In this study, using BMRT-HPV which reports type-specific viral loads/10,000 cells in the specimens, we tested only the physician-collected samples. There were 1,495 cases with adequate remaining sample from CHIMUST who were positive by Cobas, SeqHPV, and/or Cytology (≥LSIL) and 2,990 age matched, negatives in a nested case control study. (see Fig 1)

### 2.2 Study methods

#### 2.2.1 BMRT HPV PCR assay

The BMRT is a PCR-based high-risk HPV assay, which was performed with the fluorescence-based multiplex HPV DNA genotyping kit (Bioperfectus Ltd,Jiangsu, P.R. China). PCR primers and corresponding TaqMan probes were developed for the 21 most prevalent HPV types to amplify the *HPV L1* gene, including 14 HR-HPV genotype (HPV16,18,31,33,35,39, 45,51,52,56,58,59,66 and 68), and 7 MR and LR-HPV genotypes (HPV26,53,82,73,6,11 and 81). For this study, a 14 type high-risk BMRT assay was used. All types and viral loads specifically refer to high-risk HPV. To control DNA quality and determine the relative viral copy numbers in the samples, a single-copy gene encoding DNA topoisomerase III (human TOP3) was amplified in the reaction. The experimental procedure was conducted according to the kit manufacturer’s instructions. PCR was performed on an ABI Prism 7500 Fast Dx System (Thermo Fisher Scientific, Waltham, MA, USA). Five-point standard curves for HPV and the cellular log-phase were established for absolute quantification. The standard curves for HPV and TOP3 were Y = –3.85633(log10X) + 36.93833 and Y = 3.34656(log10X) + 38.51644, respectively. The number of viral copies was normalized according to the cellular input and log10-transformed Therefore, the normalization of HPV type-specific viral loads was performed as follows: viral load = log10[CnHPV/Cn TOP3) × 10,000] copies/10,000 cells, where Cn HPV is the quantity of HPV DNA and Cn TOP3 is the number of human cells. Perfectus,v1.0 was used for genotyping and quantitative analysis of HPV nucleic acids (Bioperfectus Ltd)[11].

#### 2.2.2 Cobas testing

HR-HPV testing was performed on Cobas 4800. The instrument consists of Cobas Z480 amplification analyzer and Cobas X480 automatic nucleic acid extractor (Roche Diagnostics USA). It can be used for full-automatic sample preparation and real-time PCR amplification to detect a total of 14 HR-HPV subtypes, including HPV 16, 18, 31, 33, 35, 39, 45, 51, 52, 56, 58, 59, 66 and 68, and provides the results of HPV 16 and HPV 18, and the pooled results of 12 other subtypes in the assay. All procedures were carried out in strict accordance with the working manual of the testing technology and the guidelines for the companion kit.

#### 2.2.3 Pathological diagnosis of colposcopic biopsy

Patients testing HPV positive for any HPV sample (Cobas or SeqHPV, self or direct) were called back for colposcopy, and evaluated using the POI biopsy protocol of directed and random biopsies plus endocervical curettage (ECC)[12]. Both colposcopists performed colposcopy and pathologists made histological diagnosis without knowning the results of cytology. Pathological diagnosis included negative (for intraepithelial lesion/malignancy), CIN1, CIN2, CIN3, microinvasive cancer, and invasive cancer. The highest pathological grade was taken as the final pathological diagnosis.

#### 2.2.4 Statistical analysis

SPSS 22.0 software was used for all data analysis in this study with a two-sided significance level of 0.05. Quantitative data were described by mean ± standard deviation and viral load in cervical histology was compared by One-way ANOVA. Pearson’s correlation coefficient (*r*) was applied to determine the association between viral load and cervical lesion severity. A receiver operating characteristic (ROC) curve was utilized to identify the optimal cut-point value for predicting CIN2+ using the type-specific HR-HPV viral loads. The consistency of HR-HPV between BMRT and Cobas assays was tested by Kappa coefficients. The screening efficiency of high-grade cervical lesions (CIN2+/CIN3+) was compared by McNemar test.

#### 2.2.5 Data availability

All relevant data are within the manuscript. Authors did not access to information that could identify individual participants during or after data collection.

## 3 Results

### 3.1 Overview of data

According to the study protocol, 4,485 direct-collected specimens with adequate remaining sample from the CHIMUST project were tested by the BMRT assay [1495 positives (Cobas, SeqHPV, and/or Cytology (≥LSIL) and 2990 age matched negatives]. 189 women did not have colposcopy with Cobas and/ or BMRT HR-HPV positive. 2 cases of Cobas and 1 case of cytology were technical failures. The final complete dataset of 4293 cases was analyzed, with an average age of 45.4±7.2 years. This included 748 cases of BMRT HR-HPV(+) and 810 cases of Cobas(+). The pathological diagnosis showed: normal cases (989), CIN1 (150), CIN2 (74), CIN3 (43) and cervical cancer (5). The histological results were grouped into 1139 cases of ≤CIN1 (normal and CIN1), 122 CIN2+ (CIN2, CIN3 and carcinoma), and 48 CIN3+ (CIN3 and carcinoma). (See Fig 1)

### 3.2 Correlation between type-specific HR HPV viral load and cervical lesions

Tables 1 and 2 shows that the combined viral loads of HPV16/18, 12 other subtypes HR-HPV, and all 14 HR-HPV were statistically different in all grades of cervical lesions (*P*<0.05). This was especially true for HPV16, 33 and 58 (*P*<0.01). The viral loads of HPV16/18, 12 other subtypes HR-HPV, 14 HR-HPV, HPV16, HPV33 and HPV58 increased linearly as the cervical lesions changed from ≤CIN1 to CIN3+ (HPV16/18: *r* = 0.343, *P*<0.001; other 12 subtypes HR HPV: *r* = 0.093, *P* = 0.022; 14HR HPV: *r* = 0.138, *P*<0.001; HPV16: *r* = 0.440, *P*<0.001; HPV33: *r* = 0.564, *P*<0.0001; HPV58: *r* = 0.263, *P* = 0.005).Whereas the viral load of HPV18, 31, 35, 39, 45, 51, 52, 56, 59, 66 and 68 were not predictors in all grades of cervical lesions (P>0.05).

**Table 1:** Combined 14 HR-HPV, HPV16/18, and 12 other subtypes HR-HPV viral load* relative to the grade of cervical lesion (mean±standard deviation).

Group	HPV16/18		12 other subtypes		14 HR-HPV	
	n	$\bar{\text{x}}\pm \text{s}$	n	$\bar{\text{x}}\pm \text{s}$	n	$\bar{\text{x}}\pm \text{s}$
≤CIN1	86	3.40±1.25	549	4.46±2.27	635	4.50±2.35
CIN2	20	3.74±0.88	45	4.80±1.83	65	5.01±2.39
CIN3+	34	4.37±0.67	14	5.96±2.96	48	5.81±2.68
*P* value		<0.001		0.034		<0.001

* The logarithm of HPV virus gene copy number / 10000 cells.

**Table 2:** Viral load* of individual HPV genotypes relative to the grade of cervical lesion (mean±standard deviation).

Group	HPV16		HPV18		HPV33		HPV58	
	N	$\bar{\text{x}}\pm \text{s}$	n	$\bar{\text{x}}\pm \text{s}$	n	$\bar{\text{x}}\pm \text{s}$	n	$\bar{\text{x}}\pm \text{s}$
≤CIN1	61	3.20±1.13	27	3.58±1.06	33	2.97±1.13	90	3.36±1.20
CIN2	18	3.75±0.91	2	3.63±0.76	7	4.56±1.36	16	4.53±0.89
CIN3+	33	4.32±0.79	2	3.03±2.16	2	5.54±8.27	7	4.28±0.98
*P* value		<0.001		0.792		0.001		0.01

* The logarithm of HPV virus gene copy number / 10000 cells.

### 3.3 Performance and cutoff values of BMRT viral load assay for the incidence of CIN2+ in HR-HPV infections

**Fig 2:**
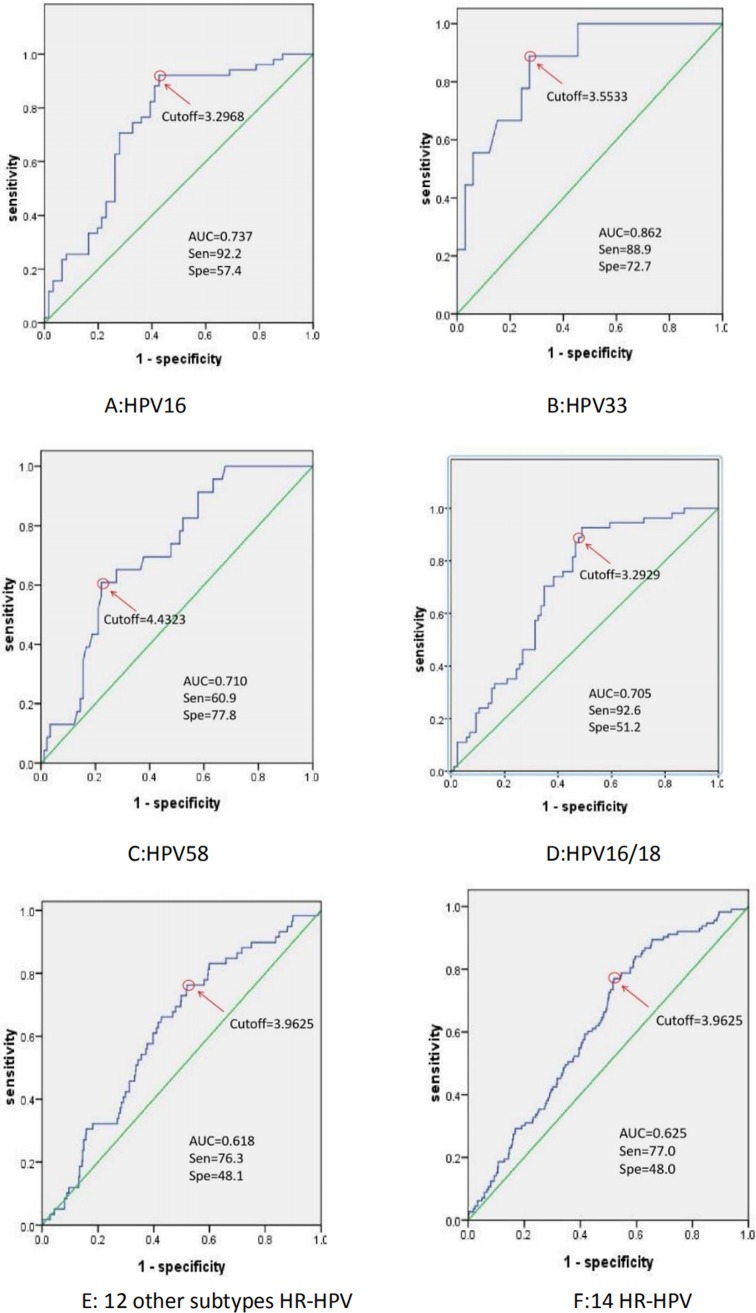
ROC curve analysis of type-specific HPV viral loads for identifying CIN2+.

We created ROC curves from the type-specific HR-HPV viral load data to determine the most useful cut-point for the identification of CIN2+ (Fig 2). According to the ROC curves the assay’s sensitivity and specificity for CIN2+ infected by HPV16/18、other 12 subtypes HR-HPV and 14 HR-HPV were 92.6%,76.3%, 77.0% and 48.0%, 51.2%, 48.1% with AUC of 0.705, 0.618, 0.625 when optimal cut-points of the log viral load achieved 3.2929, 3.9625 and 3.9625, respectively. For the individual HR-HPV viral loads, only the AUC estimates for HPV16, 33 and 58 were significant. HPV16 had an AUC of 0.737 (95% CI = 0.643, 0.830; *P*<0.001) and an optimal cut-point of 3.2968 copies/10,000 cells (Sen. = 92.2%, Spe. = 57.4%). HPV33 had an AUC of 0.862 (95% CI = 0.740, 0.983; *P*<0.001) and an optimal cut-point of 3.5533 copies/10,000 cells (Sen. = 88.9%, Spe. = 72.7%). HPV58 had an AUC of 0.710 (95% CI = 0.605, 0.815; *P*<0.001) and an optimal cut-point of 4.4323 copies/10,000 cells (Sen. = 60.9%, Spe. = 77.8%).

### 3.4 The consistency of HR-HPV subtypes between BMRT and Cobas

Table 3 showed the consistency of HR-HPV subtypes between BMRT and Cobas was 94.5% and Kappa value was 0.828.

**Table 3:** The consistency of HR-HPV subtypes between BMRT and Cobas.

BMRT	Cobas	HPV-	HPV16/18+	12 other subtypes	Consistency	Kappa
					**94.5%**	**0.828((0.804,0.849))**
**HPV-**		**3404**	**2**	**77**		
**HPV16/18+**		**31**	**137**	**17**		
**12 other subtypes**		**110**	**1**	**514**		

### 3.5 Comparison the effectiveness of BMRT and Cobas assay in cervical cancer screening

Table 4 compares a variety of BMRT HR-HPV viral load algorithms, with common Cobas and cytology strategies for primary screening and secondary screening. The parameters described were the colposcopy referral rate (%), the cytology testing rate (%), and the sensitivity and specificity for CIN2+ and CIN3+. When BMRT with it’s proprietary cut-points and Cobas were used as the primary screening strategy for cervical cancer, the sensitivity of BMRT for CIN2+ and CIN3+ was comparable to that of Cobas (92.6% vs 94.3%, 100% vs 100%, P>0.05), and its specificity was little higher than Cobas (84.8% vs 83.3%, 83.5% vs 82.0%, P < 0.001).There was no difference in coloposcopy referral rate between them(17.4% vs 18.9%, P>0.05). The triage strategy for colposcopy referral recommended by the current guidelines (algorithm #3) is Cobas HPV16/18 positive women regardless of cytology, and 12 other HPV types who have abnormal cytology. Algorithm #3 is then compared to three algorithms using BMRT-HR-HPV viral load combined subtypes without using cytology (algorithm #5, #6, #7). Compared with algorithm #3, the sensitivities of algorithms #5 and #6 for CIN2+ and CIN3+ were comparable (P>0.05); and the specificities of algorithms #5 and #6 were lower. (P<0.05). The colposcopy referral rate of algorithm #5 was comparable (P>0.05), but colposcopy referral rate of algorithm #6 was slightly increased (P<0.05). The sensitivity and specificity of algorithm #7 for CIN2+ and CIN3+ were lower than that of algorithm #3 (P<0.05); and the colposcopy referral rate of algorithm #7 was higher than that of algorithm #3.

**Table 4:** Screening algorithms.

Screening algorithms	Colposcopy(%)	Cytology (%)	CIN2+		CIN3+	
			SEN%	SPE%	SEN%	SPE%
**1**.Cobas-HPV+	18.9 (810/4293)	NA	94.3 (115/122)	83.3 (3476/4171)	100 (48/48)	82.0 (3483/4245)
**2**.BMRT-HRHPV+	17.4 (748/4293)	NA	92.6 (113/122)	84.8^a^ (3536/4171)	100 (48/48)	83.5^a^ (3545/4245)
**3**.Cobas-HPV16/18+ and cyto≥ASC-US of other subtypes	9.0a (386/4293)	14.6	81.1^a^ (99/122)	93.1^a^ (3884/4171)	100 (48/48)	92.0^a^ (3907/4245)
**4**.BMRT-HPV16/18+and cyto≥ASC-US of other subtypes	8.3#^a^ (357/4293)	14.2	81.1^a^ (99/122)	93.8*#^a^ (3913/4171)	100 (48/48)	92.7*#^a^ (3936/4245)
**5**.BMRT-HPV16/18+ and log other subtypes viral load≥3.9625	10.9*^a^ (470/4293)	NA	81.1^a^ (99/122)	91.1*#^a^ (3800/4171)	95.8 (46/48)	90.0*#^a^ (3869/4245)
**6**.BMRT-Log16/18 viral load≥3.2929, Log other subtypes viral load≥3.9625	9.8^a^ (422/4293)	NA	77.9^a^ (95/122)	92.2*^a^ (3844/4171)	95.8 (46/48)	91.1*^a^ (3869/4245)
**7**.BMRT-Log 14 HR HPV viral load≥3.9625	9.7^a^ (418/4293)	NA	71.3*#^a^ (87/122)	92.1*^a^ (3841/4171)	83.3*#^a^ (40/48)	91.1*^a^ (3868/4245)

**Abbreviations**: algorithm#1: if CobasHPV positive, refer to colposcopy;

algorithm #2: if BMRT 14 HR-HPV positive, refer to colposcopy;

algorithm #3: if Cobas HPV16/18 positive, refer to colposcopy;12 other subtypes HPV positive, underwent cytology, cytology ≥ASCUS, refer to colposcopy.

algorithm#4: if BMRT HPV16/18 positive, refer to colposcopy;12 other subtypes HR-HPV positive, underwent cytology, cytology ≥ASCUS, refer to colposcopy.

algorithm #5: if BMRT HPV16/18 positive, refer to colposcopy;12 other subtypes HR-HPV positive, determine the viral load of 12 other subtypes (the logarithm of the cut-off ≥3.9625) refer to colposcopy.

algorithm #6: if BMRT HPV16/18 positive, determine the viral load of HPV16/18 (the logarithm of the cut-off ≥3.2929) refer to colposcopy;12 other subtypes HR-HPV positive, determine the viral load of 12 other subtypes (the logarithm of the cut-off ≥3.9625) refer to colposcopy.

algorithm #7: if BMRT HR-HPV positive, determine the viral load of 14 HR-HPV (the logarithm of the cut-off ≥3.9625) refer to colposcopy.

a: compared with algorithm #1 P < 0.05;

*: when algorithms #4, #5, #6, #7 compared with algorithm #3, P < 0.05;

#: When algorithms #4, #,5 #,7 were compared with algorithm #6, P < 0.05.

## 4 Discussion

At present, the relationship between viral load and cervical lesions is still controversial. One of the important reasons for inconsistent results is that there is no standard unified high-risk HPV quantitative detection method. One of the key challenges of viral load is that sampling differences can result in varying numbers of cells in a sample. This lack of a standard has created a formidable obstacle to using viral load. BMRT can simultaneously identify the HPV type(s) and quantify the sample by testing housekeeping genes and the cells. This avoids the influence purely by the number of cells in the sample and can more accurately represents the viral load. So far there are few studies on simultaneous genotyping and viral load analysis for high-risk HPV. In a prior study we used micro-cutting technology to accurately obtain the viral load of specific subtypes of HPV in cells from cervical lesions. This work showed that HPV16/52/58 viral load was closely related with the grade of cervical lesions [7]. Dong also suggested that viral load of individual HPV16, 31, 33, 52, 58 were positively correlated with cervical lesions by using the BMRT assay [11]. A recent prospective cohort study in China studied the 10-years cumulative risk of CIN2+ in women HPV positive by the A9 group (HPV-16, -31, -33, -35, -52, - 58). Baseline positivity was 4.6%. At 10 years the cumulative risks with low and high viral loads were 16.2% and 59.2%, respectively. In contrast, no significant stratification of the women positive for the A7 group (HPV-18, -39, -45, -59, -68) was observed [8]. Our study confirmed that the viral loads of HPV16/18, 12 other subtypes HR-HPV and 14 HR-HPV were statistically different in all grades of cervical lesions (*P*<0.05), among which HPV16, 33 and 58 showed the strongest relationship. (*P*<0.01). The viral loads of HR-HPV were positively correlated with cervical lesions (*P*<0.05). It could predict that sensitivity and specificity value for CIN2+ infected by HPV16/18, 12 other subtypes HR-HPV and 14 HR-HPV were 92.6%, 76.3%, 77.0% and 48.0%, 51.2%, 48.1% when cut off values of the log viral load achieved 3.2929, 3.9625 and 3.9625, respectively. This suggests that HPV viral load is a type-specific biological indicator of cervical cancer [13–15], so when the viral load of the above HPV subtypes is greater than the corresponding cut-off values, the possibility of identifying CIN2+ is high, and it is necessary to be alert to the presence of CIN2+ lesions during colposcopy.

Li [16] confirmed the consistency of HPV infection detected by BMRT and Cobas reached 95.91%. In this study, The consistency of HR-HPV infection detected by the two assays were 94.5%, and Kappa value was 0.828 that was consistent with the above research, suggests that BMRT-HPV can be used as a screening assay for cervical cancer.

Our study shows that BMRT-HPV, as the primary screening algorithm for cervical cancer, has similar sensitivity to Cobas, which is similar to the result of the Chen’s study [17] This suggests BMRT is worthy of clinical promotion as a primary screening assay for cervical cancer.

The ideal secondary cervical cancer screening strategy should have the same characteristics as the primary screening strategy. It should be sensitive and specific for CIN 3 or cancer, reproducible, inexpensive, and not require an extensive heath care infrastructure. Admittedly, a highly specific secondary screening algorithm will, in most circumstances, compromise the overall sensitivity. In this study, the secondary screening algorithms #4,#5,#6 after BMRT-HPV primary screening have the same sensitivity for CIN3+ as Cobas primary screening, and the specificities are increased by 8%-10%, while colposcopy referral rate is also decreased significantly. This indicates that these three above secondary screening algorithms are feasible. The 2014 ASCCP transitional guidelines for cervical cancer screening. recommended HPV-16/18 typing combined with cytology as a secondary screening strategy for HPV primary screening [3]. This algorithm may need to call back patients for cytology testing depending on the screening program. Call-back will increase the cost and the rate of loss to follow-up. In addition, cytology-triage requires a highly skilled workforce and significant investment in ongoing quality assurance. This is possible and has precedent in high-income countries with the relevant infrastructure, but in the areas lacking cytology resources it is unattainable. Compared with the transitional secondary screening guidelines, “CobasHPV16/18 plus cyto≥ASCUS of 12 other types”, the secondary screening algorithms #5 and #6 “the BMRT subtypes combined viral load” have the same sensitivities for CIN2+ and CIN3+ and slightly lower specificities. The advantage of the BMRT subtypes combined viral load is that it can enable primary screening and triage to be done with one sampling and one assay and without cytology. There should be no subjectivity in HPV typing and viral load testing, so in areas lacking the resources to support a cytology infrastructure, BMRT-HPV subtypes plus viral load appears to have an advantage. There is no difference in sensitivity and colposcopy referral rate between algorithms #5 and #6, but the specificity of algorithms #6 is slightly higher than algorithms #5. Therefore, we would select algorithms #6 as our preferred secondary triage.

In this study, the BMRT test was performed on the remaining samples taken by the physician using a nested case-control design. Eight patients were BMRT HR-HPV(+) but Cobas, SeqHPV and cytology were negative, and therefore colposcopy was not performed. Considering that only 8 women who were only positive for BMRT HR-HPV were not recalled, it is believed that there is little influence on the overall data presented.

In conclusion, type-specific HR-HPV viral load is closely related to the severity of cervical lesions. BMRT HR-HPV viral load combined with subtypes can be used as a secondary strategy for HPV positive women in primary screening. This may have important application to low resource regions and we plan to test this in the future.

## Supporting information

S1 File CHIMUST protocol (English). (PDF)

S2 File CHIMUST protocol (Chinese). (PDF)
