# Supplementary material for: The application of BMRT-HPV viral load to secondary screening strategies for cervical cancer
Source: PLoS One. 2020 May 1;15(5):e0232117. doi: 10.1371/journal.pone.0232117 (PMC7194433; doi:10.1371/journal.pone.0232117)
Supplement: S1 File — (PDF) [file pone.0232117.s001.pdf]

V16B

The Chinese Multi-Center Screening Trial (CHIMUST)

<u>The Prevention of Cervical Cancer Using Self-collection as the Primary Screen</u>

Principal Investigator
Ruifang Wu MD
Professor and Chairman
Department Ob/Gyn
Peking University Shenzhen Hospital
Shenzhen, China
Wurf100@126.com

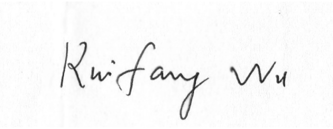

Co- PI / Study Coordinator
Jerome L Belinson MD
President, Preventive Oncology International
www.poiinc.org
Professor of Surgery, Lerner College of Medicine
Cleveland Clinic
Cleveland, Ohio, USA
jlb@poiinc.org

The Chinese Multi-Center Screening Trial (CHIMUST)

The Prevention of Cervical Cancer Using Self-collection as the Primary Screen

1. Objectives

The primary objective of the project will be to evaluate the Cobas 4800 Assay and the SeqHPV Assay for the detection of high grade pre-cancer of the uterine cervix in population based screening samples obtained using self-collection.

1.1 Secondary Objectives:

1) To evaluate differences in assay performance using solid (filter paper cards), dry brush, and liquid based specimen transport for the self-collected and physician collected cervico-vaginal specimens.
2) To evaluate cytology and genotyping as secondary screens after primary HPV screening using Cobas 4800, and SeqHPV.

2. Background and Significance

There is now significant evidence that molecular testing for (hrHPV) is more sensitive and less specific than cervical cytology for the detection of cervical precancer (CIN3 and AIS) and cancer(1-7). High-risk HPV testing is more reproducible than cytology (8,9) and with appropriate management can reduce the risk of incident cervical cancer within 4-5 years (6) and cervical cancer-related death within 8 years (7). As a consequence of its greater sensitivity for clinically important precancerous lesions, a negative hrHPV test provides a greater reassurance against incident cervical precancer and cancer than cytology (10-12), permitting safe extension of screening intervals (13).

By extension, the high sensitivity of hrHPV testing for high-grade cancer precursors and the declining level of HPV detection in normal women over the age 30 (an accepted surrogate for persistent disease) establishes testing for hrHPV as a more effective primary screen for women over 30 than cervical cytology as is currently practiced. One or two screenings in a woman's lifetime between the ages of 30 and menopause is sufficient to have a significant impact on mortality from cervical cancer (14). This now evident from multiple clinical trials. (6).

One of the advantages to switching from cytology to hrHPV testing is the possibility of using self-collected cervico-vaginal specimens, which avoids the costs and resources necessary to do a clinic-based speculum exam to obtain a cervical specimen. Self-collection and hrHPV testing has been shown to be at least as sensitive for cervical precancer and cancer as high-quality cytology (15).

We have previously demonstrated in a large population based clinical trial, The Shenzhen Cervical Cancer Screening Trial II (SHENCCAST II) that using the PCR based multiplex genotyping assay developed by BGI Shenzhen, Shenzhen, China, for mass spectrometry (MALDI-TOF-MS), a self-collected sample was equal in sensitivity to a physician obtained specimen. (16,17). These findings confirmed similar results using the Roche Linear Array Assay (18), and were supported by the

analysis of the SHENCCAST II samples using the new SeqHPV (BGI Shenzhen, Shenzhen, China)(19).

In order to apply these findings to large scale screening programs, we designed this study to use the FDA and SFDA approved Roche Cobas Assay (Roche Inc., Pleasanton CA, USA) and the new SeqHPV assay based on next generation sequencing developed by BGI Shenzhen to compare the test results from self-collected and direct collected samples and to evaluate variety specimen transportation media for each of the 2 HPV assays.

The Cobas4800 is a qualitative multiplex HPV testing assay. The test utilizes amplification of target DNA by PCR, and nucleic acid hybridization for the detection of 14 high-risk (HR) HPV types. It provides specific genotyping information for HPV types 16 and/or 18, and then pools the results for the other 12 high-risk HPV types. (20,21) Prior studies have consistently demonstrated that self-collected samples perform best when combined with a PCR based assay (16-19,22). Since the Cobas 4800 (Cobas) is both FDA and SFDA approved we feel it is important to trial the assay with self-collected samples.

The SeqHPV test is a high throughput HPV genotyping assay based on multiplex PCR and next generation sequencing. It is configured to detect 14 high risk types of HPV with a current through-put of greater than 6000 samples per day and a patient charge per sample about 1/10 other assays (~ 5 US dollars). These characteristics make it well suited for centralized laboratory processing in high volume screening programs. The assay has been previously validated using SHENCCAST II specimens. (19)

Traditionally cervical swabs have been placed in liquid media for transport. Due to the logistical difficulties such as spillage, flammability, and weight adding to the risks and costs of liquid transport media, solid carriers consisting of chemically treated or untreated filter paper have been investigated for hrHPV testing [23-34]. Solid carriers have been historically used for postnatal screening of congenital disorders [35] and more recently to detect viruses, such as HIV, and tropical diseases [36-40]. These filter paper cards are easy and safe to store and transport. Recently we (Maurer, et.al.) designed and tested a new solid media transport card that was compared to the established iFTA card from GE Healthcare. (41). The new card performed equal to the iFTA card in terms of transfer of HPV DNA and sensitivity/specificity for CIN2+. Functionally the chemistry on the card changes color with placement of the sample, lyses cells and denatures proteins upon contact, making the samples noninfectious. Importantly, the new card (POI Card), did not degrade in high humid environments like the iFTA card and it is considerably cheaper. In addition, in a sub study (Luo et. al.) provides data to suggest the iFTA may be a poorer transport vehicle than the POI card when combined with the Cobas assay. [42] Therefore for this trial we will employ the recently validated POI card, the standard PreservCyt liquid (TCT-Hologic Inc., Bedford, MA, USA). In addition we will collect “dry brush” samples from the very high-risk Inner Mongolia subset. This transport technology is of particular interest for several reasons: 1) it is very inexpensive; 2) the cards require either complex punching technology or considerable manpower upon reaching the laboratory, especially if screening programs hope to achieve high throughput; and 3) the most commonly used transport media (PreservCyt/TCT) are alcohol based and therefore restricted from routine air transport and carry some risk for home use.

For self-collected specimens the well-tested and validated split-sample methodology will be employed by using the card 1st then rinsing the brush with the residual sample in PreservCyt (TCT) transport media. (43,44).

In addition at the time of the physician visit (after the self-collection), the physician will obtain 2 “pseudo self-sample” specimens (prior to placement of a speculum). These 2 samples will be kept as “dry brush samples” by placing them in plastic bags and analyzed ≤7days and >6wks, respectively. (45)

As mentioned above, the major problem with primary HPV testing is specificity, since most of the women testing positive will have non-neoplastic HPV infections that will spontaneously clear. To address this issue we have previously investigated primary HPV screening with a mRNA assay. (46) In addition we have explored the use of 16/18 genotyping as well as multiple other HPV types for secondary triage after primary screening using both the Cervista assay and MALDI-TOF MS .(47,48) This current trial will provide the opportunity to expand that work applied to self-collection, the Cobas assay (tests for 16 and 18 separately), and SeqHPV with separate gene sequencing for 14 HPV High-risk types.

3. Study Design

3.1 <u>Methods</u>

This is a multicenter population-based cross-sectional cervical cancer-screening study that will provide screening for 10,000 women who have not been screened for cervical cancer in the past 3 years. The project will be submitted to the Peking University Shenzhen Hospital institutional review board and the IRB of the Cleveland Clinic in Cleveland, Ohio, USA for study approval. In addition the study will be registered with the Chinese Clinic Trial Registry, an international clinical trials registry platform approved by the WHO.

The clinical study consists of 1 self-sampling and 1 direct-sampling on all participants and 2 pseudo-self-sampling on participants in a high risk subset, all the samples will be used for multiple assays followed by referral for colposcopy and biopsy of women with positive test results from self-collection (any one assay), liquid direct collection (any one assay), plus pseudo-self-collection.

1) Recruitment of 10,000 patients for screening and participation in the study (Addendum flow chart)

   - Eligibility Criteria: non-pregnant, 30-59 yrs. of age, no screening for at least 3 years, no prior hysterectomy, no prior radiation, and willing to return for positive management if indicated by the primary tests.

   - <u>Exclusion Criteria</u>: Pregnant, <30 and >59 yrs of age, screening in the previous 3 years, history of a hysterectomy, pelvic radiation therapy, or not willing to return for positive management if indicated by the primary tests.

2) Informed consent

3) Demographics

4) Patient obtains 1 self-sample placed on the solid media transport card and then the brush (used as a split sample) placed and agitated in PreservCyt Liquid. The card sample will be obtained using the validated “Just for Me”™ kit, which includes a POI brush designed for both self and direct collection, a solid media specimen transport card (POI Card), and an envelope, all bar coded and contained within an outer envelope with language specific and pictorial sampling instructions. The PreservCyt sample will use the same POI brush and the sample will be placed directly into a vial containing 6ml PreservCyt.

   - # 1 sample is first rubbed on the POI Card, then the brush agitated in a 6ml PreservCyt (TCT) (the split-sample).

     These self-samples transported by card and liquid will be assayed using a) Roche-Cobas 4800 and b) BGI-SeqHPV test.

5) At ALL sites a physician will then place a vaginal speculum so as to directly-collect an endo-cervical sample from each of the participants. The sample is placed in 20ml ThinPrep® PreservCyt® (TCT) Solution (Hologic); ONLY in Inner Mongolia, prior to placement of the vaginal speculum, the physicians will obtain 2 “pseudo self-samples” which are allowed to dry, then placed into plastic bags for testing at ≤7days and >6wks respectively using a) Roche-Cobas 4800 and b) BGI-SeqHPV test.

   The direct samples will be assayed using a) Roche –Cobas and b) BGI-SeqHPV.

   Cytology using the Hologic I2 imager systems (computer assisted cytology) will used for future research not for patient care in the current study. The primary goal will be to explore secondary triage for the non-16/18, HPV positive women.

POI Card Management Protocol – Each card will be manually punched 4 times and placed in a single well in a 96-well plate. Then they are all washed once using 100 microliters sterile water. The water is carefully removed with a sterile fine-tip pipette. The DNA elution is performed in 50 microliters of sterile water at 56◦C for 30 min immediately followed by 95◦C for 15 min. in a heating block. The 96-well plate containing DNA elution and pieces of card are centrifuged at 4000 rpm for 3 min and the eluted DNA is transferred into a new 96-well plate. (Any required storage will be at -80°C for further use). When performing the assays 5ul of DNA will be used in each well of the 96 well plates for PCR for SeqHPV. 10ul will be used for a nested case control study using Cobas. (It should be noted that this is the standard volume used for SeqHPV, and it has been thoroughly tested and demonstrated to be optimal with > 99% adequate specimens. It was determined in our prior study that 10ul optimized the Cobas Assay when using the POI card.)

Flow Chart with assays (2 analytics nested):

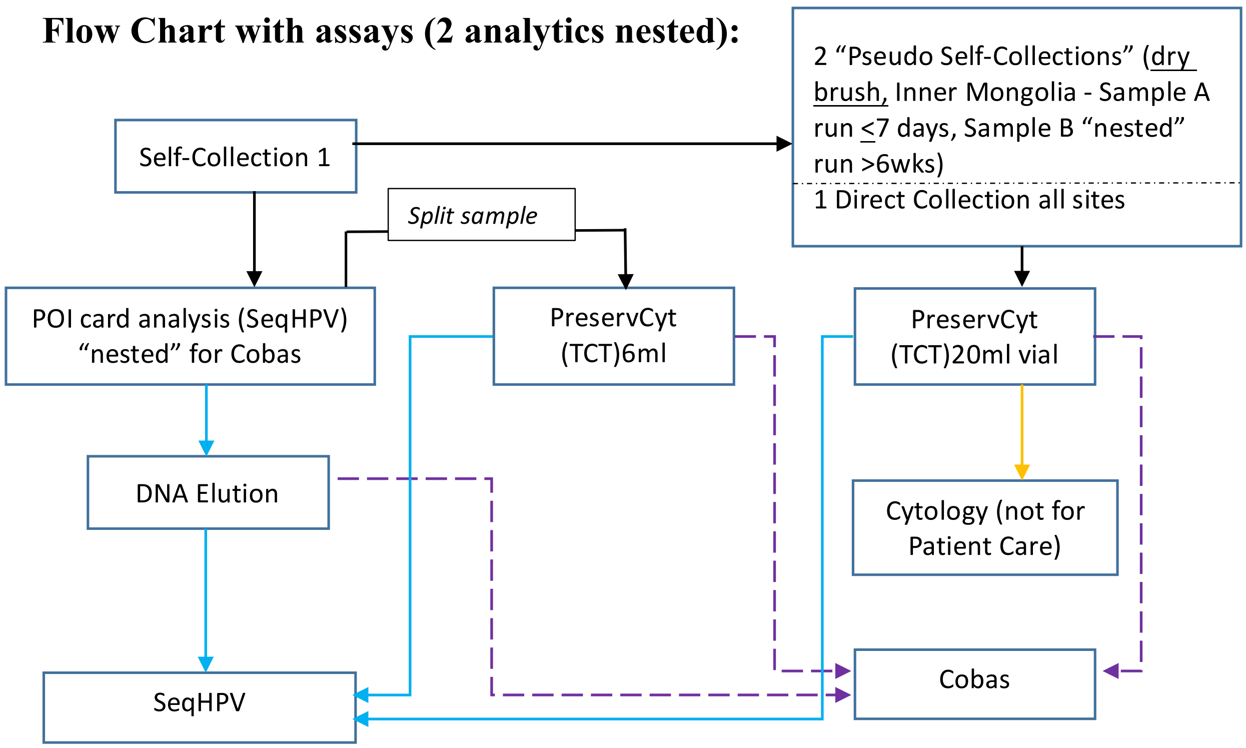

Sample applications:

1 Self-collection ------to ❶POI Card ---- then Split Sample into ❷ PreservCyt (6ml)

2 Physician pseudo self-collections – (Inner-Mongolia site only) ❸ Dry Brushes stored Dry for analysis, **Sample A** ≤7 days; **Sample B** nested analysis >6wks

1 Physician Direct endocervical collection --------to ❹PreservCyt (20ml)

Assay:

From the ❶POI Card -----All SeqHPV (5ul HPV DNA); *nested* Cobas (10ul HPVDNA)

From the ❷ PreservCyt (6ml) --------Cobas (1ml), SeqHPV(1ml)

From the ❸ Sample A (reconstituted) ---- Cobas (1ml), SeqHPV (1ml)

From the ❸ Sample B (reconstituted) -----*nested* Cobas (1ml), SeqHPV (1ml)

From the ❹PreservCyt(20ml)--------Cobas (1ml), SeqHPV(1ml), and Cytology.

6) Patients testing hrHPV positive from any of ❶- to ❹ will be recalled for colposcopy using the POI protocol of directed and/or random biopsies plus ECC (49).

7) Patient management will be determined based on biopsy results and according to current standards at the Peking University Shenzhen Hospital.

3.2 Study Population:

Ten thousand women meeting the enrollment criteria will be recruited for this study from five sites in China, which are selected from 1) Beijing, 2) Shanghai, 3) Shijiazhuang, Hebei Province, 4) Wuhan, Hubei Province, and 5) Wushen County, Erdose, Inner Mongolia.

The enrollment period is expected to begin June 1st, 2016, and be completed by December 31st, 2016. All patient management will be completed by March 31st, 2017.

The study PI intends to give preference to “rural” women. This is a very important distinction in China, since “rural” families who have migrated to the city often find they are outsiders to the economy including the education and healthcare systems.

The research sites are selected to represent: 1) the developed, developing and the undeveloped areas in in both the South and North of China. 2) The areas with rural populations recently urbanized and/or still agricultural. 3) Areas with medical resources varying from sufficient to insufficient (medically underserved)

Shanghai was selected because 1) it is one of the most developed cities in China, representing an economically developed area in Southeast China; 2) there is a large recently urbanized rural population; and 3) its per-capital medical resources are among the highest.

Beijing was selected because 1) it is one of the most developed cities in China representing an economically developed area in Northeast China; 2) there is a large recently urbanized rural population and agricultural population; and 3) its per-capital medical resources are among the highest.

Wuhan, the capital of Hubei Province, was selected because 1) it is a typical developing city located in central China and represents the largest settlement of Chinese living in the Yangzi River basin; 2) it has large rural/agricultural population; and 3) it is one of the second line cities with insufficient medical resources.

Shijiazhuang, the capital of Hebei Province, was selected because 1) it is a typical developing city located in Northern China; 2) it has a large rural/agricultural population; 3) it is one of the second line cities with insufficient medical resources.

Wushen County in Erdose, one of the cities in Inner Mongolia Autonomous Administrative Region, was selected because 1) it is one of the undeveloped cities in Northwest China with a

large agricultural population; 2) the HPV infection rate has been reported to be 22%; 3) the majority of the population are rural people working in agriculture and grassland farming; and 4) it is one of the fourth-line cities in China with insufficient (very poor) medical resources.

3.3 Data Management and Statistical Methods:

Data Management

A strict system of data management will be utilized. All participating women will be assigned a study ID number upon entry into the study. Only the Principal Investigator and Project Coordinators will have access to the complete files matching personal identifiers to the confidential study ID numbers. Laboratory samples and results are identified only by confidential code numbers with no

available personal identifiers. Laboratory information that could in any way be linked to a particular study participant by name or personal identifier will not be released except to provide that patient with needed medical care and appropriate follow-up care. All reports, publications and scientific presentations of the findings from the study will be absent of any personal identifiers, and therefore presented without the identities of individual participants.

Samples are tracked and the study questionnaire and laboratory data will be entered onto the study data forms at the clinical sites. The forms will be copied and the original copy transferred to the data management office at Peking University Shenzhen Hospital to be dual entered into the Study Data Management System. These files will be backed up nightly. All forms will be stored in locked files by the Project Coordinator.

Data management will include quality control procedures including the employing of range checks and assessment of completeness and consistency across variables. The site specific data manager will be supervised by the study assigned epidemiologist and biostatistician. All data will be merged into one cleaned and locked database to create a final set for data analysis.

Data Analysis

Data analysis will be a collaboration between Preventive Oncology International, affiliated with the Women's Health Institute, the Cleveland Clinic in Cleveland, Ohio, USA and Peking University Shenzhen Hospital, Shenzhen, China.

Statistical Methods

The primary endpoints of our study will be the sensitivity and specificity of the hrHPV tests for cervical intraepithelial neoplasia (CIN) 3 or cancer (CIN 3+). The secondary endpoints will be the sensitivity and specificity of the hrHPV tests for CIN 2+.

Our estimates of the difference in sensitivity for CIN 3+ and specificity for CIN 3+ that may be detected are based on the prevalence of hrHPV and prevalence of CIN 3+ (the primary endpoint) or CIN 2+ (the secondary endpoint) in the population studied. Our estimates of the prevalence of hrHPV and CIN 3+ are likely more accurate than in many studies as we can use the data from SHENCCAST II (17) as a guide. In SHENCCAST II (17), we screened populations similar to four of our study sites; and our fifth site (Inner Mongolia) has known hrHPV rates and resulting pre-invasive/invasive cervical cancer considerably higher than any sites in SHENCCAST II. Adding the site in Inner Mongolia should compensate for the age difference of 25-59 years (SHENCCAST II) vs. 30-59 years for the current trial (CHIMUST).

As in SHENCCAST II, sensitivities and specificities will be calculated for each hrHPV tests. Logistic regression analysis will be used to generate odds ratios and 95 % CI for factors that may be associated with test results by sampling device.

Sample size calculations to compare the sensitivity and specificity of hrHPV tests are based on the prevalence of hrHPV and the prevalence of the endpoint (CIN 3+ or CIN 2+) in the population. In SHENCCAST II, the prevalence of hrHPV measured in vaginal self-collected or endocervical practitioner-collected samples and tested for hrHPV by MALDI-TOF or Cervista assays was between 11.1% and 14.8%. The prevalence of the primary endpoint of CIN 3+ in SHENCCAST II

was 1.6% (141/8,556) while that of the secondary endpoint of CIN 2+ was 2.7% (227/8,556). The major results from SHENCCAST II are reproduced in Table 1 below. The SeqHPV assay being used in this trial, was trained and tested on SHENCCAST II specimens and showed results similar to MALDI-TOF. (19)

Table 1: Comparison of the sensitivity and specificity for ≥CIN 3 of vaginal self-collected and physician collected specimens (endocervical) assayed for HR-HPV by Cervista and MALDI-TOF.

(95% confidence intervals and the actual patient numbers are in parentheses). McNemars p-values for the comparison of the sensitivity of self-collected MALDI-TOF to physician collected Cervista is 1.0. McNemars p-value for the comparison for the specificities is p=<0.0001

SPECIMEN/HR-HPV TEST	SENSITIVITY FOR ≥CIN 3 (%) (C.I.)(n)	SPECIFICITY FOR ≥CIN 3 (%) (C.I.)(n)
Vaginal self-collected / Cervista	**70.9%** (62.7-78.3) (100/141)	**86.1%** (85.3-86.8) (7248/8415)
Endocervical / Cervista	**95.0%** (90.0-98.0) (134/141)	**90.3%** (89.6-90.9) (7598/8415)
Vaginal self-collected / MALDI-TOF	**94.3%** (89.1-97.5) (133/141)	**87.5%** (86.8-88.2) (7370/8415)
Endocervical / MALDI-TOF	**94.3%** (89.1-97.5) (133/141)	**89.4%** (88.7-90.0) (7526/8415)

The 95% CI for the sensitivity of hrHPV in Table 1 spans about 8%, suggesting that, with a study involving 10,000 women with prevalence of hrHPV of 11.1 to 14.8% and prevalence of CIN 3+ of 1.6%, we can reliably detect a difference in sensitivity of about 8%. The 95% CI for the specificity of hrHPV in Table 1 spans only about 1.3% suggesting that we can reliably detect differences in specificity of the hrHPV test greater than 1.3%. As we have paired specimens, the difference in sensitivity for CIN 3+ that can reliably be detected is closer to 7% i.e. 94.3% (133/141) vs. 87.2% (123/141) rather than 8%.

<u>Primary Objective:</u> *Evaluation of the Cobas 4800 Assay, and the SeqHPV Assay for the detection of high grade pre-cancer of the uterine cervix, in population based screening samples obtained using self-collection.*

To evaluate the comparative sensitivities and specificities of the hrHPV platforms, the sensitivity and specificity of each test will be calculated using biopsy showing CIN 3+ (primary endpoint) or CIN 2+ (secondary endpoint) as the gold standards.

<u>Secondary objective 1:</u> *To evaluate differences in assay performance using solid (filter paper cards), dry brush, and liquid based specimen transport for the self-collected and physician collected cervico-vaginal specimens. .*

To determine which kind of sample collection media is a suitable medium for collecting and storing vaginal self-samples relative to the assay used we will calculate sensitivities for detecting CIN2+

and CIN3+ and the number of insufficient samples for hrHPV testing of the different sample collection transport systems.

Secondary objective 2: *To evaluate cytology and genotyping as secondary screens after primary hrHPV screening using Cobas and SeqHPV.*

The focus will be to explore practical algorithms that will maximize specificity for CIN 3+ with minimal loss in sensitivity for CIN 3+. Differences in sensitivity and specificity for CIN 3+ will be compared with McNemar's test when there are paired results and Chi-Square when there are independent groups. Cytology will be analysed as a triage technology with a special focus on the non-HPV 16/18 positive population. For the genotyping analysis the available types (14 HPV types) will be used from SeqHPV. Types 16/18 will be used from SeqHPV and Cobas.

4. Patient Safety:

This is a non-interventional study. In this study, only cervical/vaginal samples will be collected. The occurrence of AEs / SAEs will therefore be considered unrelated to the diagnostic tests. Therefore, no indirect harm related to the investigated diagnostic tests is anticipated in the context of this study.

5. Compliance Statement:

Study approval by the IRBs of the Cleveland Clinic and Peking University Shenzhen Hospital meets all of the elements covered by the following compliance statement:

> *The study will be conducted in compliance to this study protocol, the current version of the Declaration of Helsinki, ICH GCP, and applicable local legal and regulatory requirements.*
>
> *Submission of study documents: Before study start, the study protocol, and subject information / informed consent and any other study-related document as required by applicable laws and regulations will be submitted to the Ethics Committee and regulatory authorities for written approval. Any protocol amendments or new or amended information that requires ethical consideration will be submitted for written approval, too. In addition, a study report (interim and /or full report) will be submitted to regulatory authorities in line with applicable timelines.*
>
> *Subject information and informed consent: Subjects/legal representatives will be informed orally and in writing about the objectives of the study, study procedures, potential risks, and about the fact that to some extent data will be accessible for third parties (see below) for the purpose of controlling the study conduct - provided that data confidentiality is ensured at any time.*
> *Before any study-related activities are initiated, the subjects / legal representatives will have to sign the written informed consent. The participation in the study is entirely voluntary. The subjects have the right to withdraw their willingness to participate in the study at any time without affecting their future medical care in any way.*
>
> *Clinical study results and publication: The results of the clinical study will be documented in a clinical study report and if possible, will be published (e.g. in a journal or presented in a scientific meeting).*

References

1. Cuzick J, Clavel C, Petry KU, Meijer CJ, Hoyer H, Ratnam S, Szarewski A, Birembaut P, Kulasingam S, Sasieni P, Iftner T. Overview of the European and North American studies on HPV testing in primary cervical cancer screening. Int J Cancer. 2006;119(5):1095-101.
2. Mayrand MH D-FE, Rodrigues I,Walter SD, Hanley J, Ferenczy A, Ratnam S,Coutlee F,Franco EL; cervical cancer screening trial study group. Human Papillomavirus DNA versus Papanicolaou screening tests for cervical cancer. N Engl J Med. 2007;357(16):1579-88.
3. Naucler P, Ryd W, Tornberg S, Strand A, Wadell G, Elfgren K, Radberg T, Strander B, Johansson B, Forslund O, Hansson BG, Rylander E, Dillner J. Human papillomavirus and Papanicolaou tests to screen for cervical cancer. N Engl J Med. 2007;357(16):1589-97.
4. Ronco G, Giorgi-Rossi P, Carozzi F, Confortini M, Dalla Palma P, Del Mistro A, Ghiringhello B, Girlando S, Gillio-Tos A, De Marco L, Naldoni C, Pierotti P, Rizzolo R, Schincaglia P, Zorzi M, Zappa M, Segnan N, Cuzick J, New Technologies for Cervical Cancer screening Working G. Efficacy of human papillomavirus testing for the detection of invasive cervical cancers and cervical intraepithelial neoplasia: a randomised controlled trial. Lancet Oncol. 2010;11(3):249-57.
5. Rijkaart DC, Berkhof J, Rozendaal L, van Kemenade FJ, Bulkmans NW, Heideman DA, Kenter GG, Cuzick J, Snijders PJ, Meijer CJ. Human papillomavirus testing for the detection of high-grade cervical intraepithelial neoplasia and cancer: final results of the POBASCAM randomised controlled trial. Lancet Oncol. 2012;13(1):78-88.
6. Ronco G, Dillner J, Elfstrom KM, Tunesi S, Snijders PJ, Arbyn M, Kitchener H, Segnan N, Gilham C, Giorgi-Rossi P, Berkhof J, Peto J, Meijer CJ, the International HPVswg. Efficacy of HPV-based screening for prevention of invasive cervical cancer: follow-up of four European randomised controlled trials. Lancet. 2013.
7. Sankaranarayanan R, Nene BM, Shastri SS, Jayant K, Muwonge R, Budukh AM, Hingmire S, Malvi SG, Thorat R, Kothari A, Chinoy R, Kelkar R, Kane S, Desai S, Keskar VR, Rajeshwarkar R, Panse N, Dinshaw KA. HPV screening for cervical cancer in rural India. N Engl J Med. 2009;360(14):1385-94.
8. Castle PE, Wheeler CM, Solomon D, Schiffman M, Peyton CL, Group A. Interlaboratory reliability of Hybrid Capture 2. Am J Clin Path. 2004;122(2):238-45.
9. Carozzi FM, Del Mistro A, Confortini M, Sani C, Puliti D, Trevisan R, De Marco L, Tos AG, Girlando S, Palma PD, Pellegrini A, Schiboni ML, Crucitti P, Pierotti P, Vignato A, Ronco G. Reproducibility of HPV DNA Testing by Hybrid Capture 2 in a Screening Setting. Am J Clin Path. 2005;124(5):716-21.
10. Dillner J, Rebolj M, Birembaut P, Petry KU, Szarewski A, Munk C, de Sanjose S, Naucler P, Lloveras B, Kjaer S, Cuzick J, van Ballegooijen M, Clavel C, Iftner T, Joint European Cohort S. Long term predictive values of cytology and human papillomavirus testing in cervical cancer screening: joint European cohort study. BMJ. 2008;337:a1754.

11. Andrae B, Kemetli L, Sparen P, Silfverdal L, Strander B, Ryd W, Dillner J, Tornberg S. Screening-preventable cervical cancer risks: evidence from a nationwide audit in Sweden. J Natl Cancer Inst. 2008;100(9):622-9.
12. Shi JF, Belinson JL, Zhao FH, Pretorius RG, Li J, Ma JF, Chen F, Xiang W, Pan QJ, Zhang X, Zhang WH, Qiao YL, Smith JS. Human papillomavirus testing for cervical cancer screening: results from a 6-year prospective study in rural China. Am J of Epidemiol. 2009;170(6):708-16.
13. Saslow D, Solomon D, Lawson HW, Killackey M, Kulasingam SL, Cain J, Garcia FA, Moriarty AT, Waxman AG, Wilbur DC, Wentzensen N, Downs LS, Jr., Spitzer M, Moscicki AB, Franco EL, Stoler MH, Schiffman M, Castle PE, Myers ER, Committee A-A-ACCG. American Cancer Society, American Society for Colposcopy and Cervical Pathology, and American Society for Clinical Pathology screening guidelines for the prevention and early detection of cervical cancer. CA: a cancer journal for clinicians. 2012;62(3):147-72.
14. Kim JJ, Brisson M, Edmunds WJ, Goldie SJ. Modeling cervical cancer prevention in developed countries. Vaccine. 2008 Aug 19;26 Suppl 10:K76-86. doi: 10.1016/j.vaccine.2008.06.009.
15. Zhao FH, Lewkowitz AK, Chen F, Lin MJ, Hu SY, Zhang X, Pan QJ, Ma JF, Niyazi M, Li CQ, Li SM, Smith JS, Belinson JL, Qiao YL, Castle PE. Pooled analysis of a self-sampling HPV DNA Test as a cervical cancer primary screening method. J Natl Cancer Inst. 2012;104(3):178-88.
16. Belinson JL, Du H, Yang B, et al. Improved sensitivity of vaginal self-collection and high-risk human papillomavirus testing. Int J Cancer 2012; 130:1855-1860.
17. Du H, Yi J, Wu R, et al. A New PCR based Mass Spectrometry System for High-Risk HPV Part II – Clinical Trial. Am J Clin Pathol.2011;136:920-923
18. Belinson JL, Hu S, Niyazi M, et al. Prevalence of type-specific human papillomavirus in endocervical, upper and lower vaginal, perineal, and vaginal self-collected specimens; implications for vaginal self-collection. Int J Gyn Cancer 2010 Sept 1;127:1151-1157.
19. Yi X, Zou J,Xu J, et al. Development and validation of a new HPV genotyping assay based on next generation sequencing. Am J Clin Pathol. 2014;141:796-804.
20. Wright TC, Stoler MH, Behrens CM, Sharma A, Zhang G, Wright TL. Primary cervical cancer screening with human papillomavirus: end of study results from the ATHENA study using HPV as the first-line screening test. Gynecol Oncol. 2015 Feb;136(2):189-97. doi: 10.1016/j.ygyno.2014.11.076. Epub 2015 Jan 8.
21. Castle PE, Stoler MH, Wright TC Jr, Sharma A, Wright TL, Behrens CM. Performance of carcinogenic human papillomavirus (HPV) testing and HPV16 or HPV18 genotyping for cervical cancer screening of women aged 25 years and older: a subanalysis of the ATHENA study. Lancet Oncol. 2011 Sep;12(9):880-90. doi: 10.1016/S1470-2045(11)70188-7. Epub 2011 Aug 22.
22. Arbyn M, Verdoodt F, Snijders PJ, Verhoef VM, Suonio E, Dillner L, Minozzi S, Bellisario C, Banzi R, Zhao FH, Hillemanns P, Anttila A. Accuracy of human papillomavirus

testing on self-collected versus clinician-collected samples: a meta-analysis. Lancet Oncol. 2014 Feb;15(2):172-83. doi: 10.1016/S1470-2045(13)70570-9. Epub 2014 Jan 14.
23. Kailash U, Hedau S, Gopalkrishna V, Katiyar S, Das BC. A simple 'paper smear' method for dry collection, transport and storage of cervical cytological specimens for rapid screening of HPV infection by PCR. J Med Microbiol. 2002 Jul;51(7):606-10.
24. Gustavsson I, Lindell MF, Wilander EF, Strand AF, Gyllensten U. Use of IFTA card for dry collection, transportation and storage of cervical cell specimen to detect high-risk HPV. J Clin Virol. 2009: 46: 112-6.
25. Gustavsson I, Sanner KF, Lindell MF, Strand AF, Olovsson MF, Wikstrom IF, et al. Type-specific detection of high-risk human papillomavirus (HPV) in self-sampled cervicovaginal cells applied to iFTA elute cartridge. J Clin Virol 2011; 51:255-8.
26. Lenselink CH, de Bie RP, van Hamont D, Bakkers JM, Quint WG, Massuger LF, Bekkers RL, Melchers WJ: Detection and genotyping of human papillomavirus in self-obtained cervicovaginal samples by using the FTA cartridge: new possibilities for cervical cancer screening. J Clin Microbiol 2009, 47:2564–70.
27. De Bie, R.P., Schmeink CE, Bakker JM, Snijder PJ, Quint WG, Massuger, L.F., Bekkers, R.L., Melchers, W.J., 2011. The indicating FTA elute cartridge a solid sample carrier to detect high-risk HPV and high-grade cervical lesions. J. Mol. Diagn. 2011, 13: 371–6.
28. Gonzalez P, Cortes B, Quint W, Kreimer AR, Porras C, Rodriguez AC, et al. Evaluation of the IFTA carrier device for human papillomavirus testing in developing countries. J Clin Microbiol. 2012 Dec; 50(12):3 870-6.
29. Geraets DT, van Baars R, Alonso I, Ordi J, Torne A, Melchers WJ, et al. Clinical evaluation of high-risk HPV detection on self-samples using the indicating FTA-elute solid-carrier cartridge. J Clin Virol. 2013 Jun; 57(2):125-9.
30. Guan Y, Gravitt PE, Howard R, Eby YJ, Wang S, Li B, et al. Agreement for HPV genotyping detection between self-collected specimens on a iFTA cartridge and clinician-collected specimens. J Virol Methods. 2013 Apr; 189(1):167-71.
31. Wang SM, Hu SY, Chen F, Chen W, Zhao FH, Zhang YQ, et al. Clinical evaluation of human papillomavirus detection by careHPV test on physician-samples and self-samples using the indicating FTA elute card. Asian Pac J Cancer Prev. 2014;15(17):7085-9.
32. Gyllensten U, Gustavsson I, Lindell M, Wilander E. Primary high-risk HPV screening for cervical cancer in post-menopausal women. Gynecol Oncol. 2012 May;125(2):343-5.
33. Phongsavan K, Gustavsson I, Marions L, Phengsavanh A, Wahlstrom R, Gyllensten U. Detection of human papillomavirus among women in laos: Feasibility of using filter paper card and prevalence of high-risk types. Int J Gynecol Cancer. 2012 Oct;22(8):1398-406.
34. Santos CR, Franciscatto LG, Barcellos RB, Almeida SE, Rossetti ML. Use of FTA elute card impregnated with cervicovaginal sample directly into the amplification reaction increases the detection of human papillomavirus DNA. Braz J Microbiol. 2012 Jan;43(1):389-92.

35. Mei JV, Alexander JR, Adam BW, Hannon WH. Use of filter paper for the collection and analysis of human whole blood specimens. J Nutr. 2001 May;131(5):1631S-6S.
36. Beebe JL, Briggs LC. Evaluation of enzyme-linked immunoassay systems for detection of human immunodeficiency virus type 1 antibody from filter paper disks impregnated with whole blood. J Clin Microbiol. 1990 Apr;28(4):808-10.
37. Ayele W, Schuurman R, Messele T, Dorigo-Zetsma W, Mengistu Y, Goudsmit J, et al. Use of dried spots of whole blood, plasma, and mother's milk collected on filter paper for measurement of human immunodeficiency virus type 1 burden. J Clin Microbiol. 2007 Mar;45(3):891-6.
38. Fiscus SA, Cheng B, Crowe SM, Demeter L, Jennings C, Miller V, et al. HIV-1 viral load assays for resource-limited settings. PLoS Med. 2006;3(10):e417.
39. Pitcovski J, Shmueli E, Krispel S, Levi N. Storage of viruses on filter paper for genetic analysis. J Virol Methods. 1999;83(1-2):21-6.
40. Smit PW, Elliott I, Peeling RW, Mabey D, Newton PN. An overview of the clinical use of filter paper in the diagnosis of tropical diseases. Am J Trop Med Hyg. 2014 Feb;90(2):195-210.
41. Maurer K , Luo H, Shen Z, Wang G, Wang G, Du H, Qu X, Wu R, and Belinson J. The Development and Evaluation of a New Solid Media Specimen Transport Card for Population Based Cervical Cancer Prevention. (Manuscript in preparation as of 6-19-2015).

42. Hongxue Luo, Hui Du, Kathryn Maurer, Jerome L.Belinson, Guixiang Wang, Zhihong Liu, Lijie Zhang, Yanqiu Zhou, Chun Wang, Jinlong Tang, Xinfeng Qu, Ruifang Wu. An evaluation of the Cobas4800 HPV test on cervico-vaginal specimens in liquid versus solid transport media. Accepted Jan. 2016, Plos One.

43. Biscotti CV, O'Brien DL, Gero MA, Gramlich TL, Kennedy AW, Easley KA: Thin-layer Pap test vs. conventional Pap smear: Analysis of 400 split samples. J Reprod Med 2002;47:9-13.
44. Hutchinson ML, Agarwal P, Deanult T, Berger B, Cibas ES: A new look at cervical cytology: ThinPrep multicenter trial results. Acta Cytol 1992;36:499-504.
45. Rosa Catarino, Pierre Vassilakos, Aline Bilancioni, Mathieu Vanden Eynde, Ulrike Meyer-Hamme, Pierre-Alain Menoud, Frederic Guerry, Patrick Petignat. Self-sampling methods for human papillomavirus detection: dry swabs versus FTA cartridge.PLOS One; doi:10.1371/journal.pone.0143644. Dec.2,2015
46. Wu R, Belinson SE, Du H, et al. Human papillomavirus (HPV) mRNA assay for cervical cancer screening:The Shenzhen Cervical Cancer Screening Trial I (SHENCCAST I). Int J Gynecol Cancer. 2010;20:1411-1414.
47. Wu R, Du H, Belinson SE, et al. Secondary screening after primary self-sampling for human papillomavirus from SHENCCAST II. J Low Genit Tract Dis. 2012;16:416-420.
48. Goodrich SK, Pretorius RG, Du H, et al. Triage of women with negative cytology and positive high-risk HPV: An analysis of data from the SHENCCAST II/III studies. J Low Genit Tract Dis. 2014;18:122-127.

49. Belinson JL, Qiao YL, Pretorius RG, Zhang WH et al: Shanxi Province Cervical Cancer Screening Study. A Cross-Sectional Comparative Trial of Multiple Techniques to Detect Cervical Intraepithelial Neoplasia. Gynecol Oncol 83: 439-44, Nov 2001.
