# Supplementary material for: The application of BMRT-HPV viral load to secondary screening strategies for cervical cancer
Source: PLoS One. 2020 May 1;15(5):e0232117. doi: 10.1371/journal.pone.0232117 (PMC7194433; doi:10.1371/journal.pone.0232117)
Supplement: S2 File — (PDF) [file pone.0232117.s002.pdf]

第 16B 版

中国宫颈癌筛查研究

自取样 HPV 检测为宫颈癌初筛方案的多中心研究

The Chinese Multi-Center Screening Trial. CHIMUST

The Prevention of Cervical Cancer Using Self-collection as the Primary Screen

项目负责人

吴瑞芳教授

北京大学深圳医院妇产科主任

wurf100@126.com

共同负责人/ 研究协调人员

Jerome L Belinson 教授

国际防癌组织主席（**Preventive Oncology International，POI**）

www.poiinc.org

美国克利夫兰医学中心、**Lerner** 医学院外科教授

jlb@poiinc.org

中国宫颈癌筛查研究(CHIMUST)

自取样 HPV 检测为宫颈癌初筛方案的多中心研究

The Chinese Multi-Center Screening Trial (CHIMUST)

The Prevention of Cervical Cancer Using Self-collection as the Primary Screen

1. 研究目的

本研究的主要研究目的是：评价 Cobas 4800 HPV 检测技术（简称 Cobas）和 SeqHPV 检测技术自人群筛查中获取的阴道自取样本中检出宫颈高度病变（癌前病变）的效果。

本研究的次级目标是:

1) 评价不同检测方法对固体（滤纸卡）、干刷、和液体样本保存转运介质的阴道自取样和医生取样样本的检测效果;
2) 评价细胞学和 Cobas4800 和 SeqHPV 检测方法初筛后，以细胞学和 HPV 亚型作为二次筛查的效果。

2. 研究背景及意义

目前已有重要的证据证实，针对高危型 HPV（High-risk HPV，hr-HPV）的分子生物学检测在诊断宫颈癌前病变(CIN3 和 AIS)或宫颈癌方面相对于细胞学方法敏感性更高，但特异性较低[1-7]。高危型 HPV 检测比细胞学重复性好[8,9]，只要采用恰当的阳性患者管理流程，HPV 检测可以降低 4-5 年内宫颈癌的发生风险[6]和 8 年内宫颈癌相关的死亡风险[7]。由于其诊断宫颈癌癌前病变的敏感性很高，hrHPV 检测阴性可以提供比细胞学正常更高的不发生宫颈癌前病变和宫颈癌的保证[10-12]，并允许更长的安全筛查间隔[13]。

高危型 HPV 检测对诊查高度宫颈癌前病变的高敏感性和 30 岁以上女性的 HPV 感染水平的下降趋势（一个标志持续感染可接受的标志）使得 hrHPV 检测完全可以替代细胞学，成为 30 岁以上女性宫颈癌筛查的更为有效的初筛手段。在女性一生中，于 30 岁到绝经期间进行 1-2 次 HPV 检测的筛查，就足以对宫颈癌死亡率产生重大影响[14]。这一结论目前得到了多项临床试验证据的支持[6]。

宫颈癌筛查从细胞学检查切换到 hrHPV 检测的优点之一是可以采用宫颈/阴道自取样本。自取样避免了临床医生以妇科检查方式收集宫颈脱落细胞样本所必需的成本。研究证实：采用阴道自取样样本进行 hrHPV 检测诊断宫颈癌癌前病变和宫颈癌至少具有和高质量细胞学检查同样的敏感性[15]。

在“深圳宫颈癌筛查项目 II”（SHENCCAST II）这一大样本临床试验项目中，我们已经证实，采用由深圳华大基因研究院（中国深圳）开发的多重 PCR 技术加质谱平台（MALDI-TOF-MS）基因分型检测，自取样样本和医生取样样本具有相同的检测敏感性[16,17]。这一发现进一步确认了采用 Roche Linear Array Assay 检测技术所得到的类似结果[18]，并得到了以华大基因研究院开发的 SeqHPV 对 SHENCCAST II 样本进行检测分析所得到的结果的支持[19]。

为了将这些研究成果应用于大规模人群筛查，我们设计了本研究方案，目的是应用已获得 FDA 和 SFDA 批准的 Cobas HPV 检测 (Roche Inc., Pleasanton CA, USA)和深圳华大基因研究院开发的基于二代测序技术的 SeqHPV 检测，对自取样样本和医生取样样本的检测结果进行比较，并对多种保存方式在每种检测平台的应用进行评价。

Cobas 是一种多重 HPV 定性检测。该检测采用 PCR 技术扩增靶物 DNA，然后以核酸杂交检测 14 种高危型 HPV。Cobas 提供 HPV16 和/或 HPV18 基因分型的报告，并将其它 12 个高危型别 HPV 混合在一起报告[20,21]。既往的多项研究均证实，可使阴道自取样样本获得最佳检测效果的是 PCR 平台的检测[16-19,22]，鉴于 Cobas 已通过了 FDA 和 SFDA 两项认证，我们认为验证它对阴道自取样样本的检测效果非常重要。

SeqHPV 检测是基于多重 PCR 和二代测序平台的高通量 HPV 基因分型检测技术。这项检测可检测到 14 种 HPV 高危型别，目前的检测通量是每日 6000 例以上，单例检测费用是其它 HPV 检测方法的 1/10 左右（约 5 美元）。这些特性决定了这项检测非常适合大规模筛查时的中心实验室集中检测。我们曾利用 SHENCCAST II 样本对这项检测进行过验证[19]。

传统的宫颈取样刷一直是放置在液体介质中转运的。由于液体溢出、易燃以及重量等物流问题增加了运输风险和花费，人们开始对以经过或未经过化学处理的滤纸制造的固体转运介质用于 HPV 检测进行研究[23-24]。固体样本介质一直被用于新生儿出生缺陷的筛查[35]，最近也开始用于病毒（如 HIV）和热带病检测[36-40]。这些滤纸的储存和运输非常容易和安全。最近我们(Maurer 等)设计并验证了一款新的固体介质转运卡（POI 卡），并将其与已经验证过的 iFTA 卡（来自 GE Healthcare）进行了比较[41]。POI 卡和 FTA 卡在 HPV DNA 转运量和诊断 CIN2+的敏感性和特异性方面效果相同。从功能上讲，POI 卡上的化学物质具有在涂布样本后产生颜色变化、裂解细胞核降解蛋白质的作用，从而使涂布其上的样本失去传染性。更重要的是，POI 卡不会像 iFTA 卡那样，在潮湿环境中降解，而且价格相当便宜。此外，这项试验的一次附加试验（LUO 等）所提供的数据显示，当 iFTA 卡被用于 Cobas 检测时，其作为样本转运媒介的功能逊于 POI 卡[42。因此，在本研究中，我们将同步采用新近验证的 POI 卡和标准的 PreservCyt 保存液（TCT-Hologic Inc., Bedford, MA, USA） 作为样本转运介质。此外，我们还将在高风险的内蒙古筛查现场采集“干刷样本”。干刷样本受到广泛关注是因为 1）干刷样本保存极其廉价；2）取样卡依然存在在实验室应用中的打孔复杂或人力投入较大等问题，且在大型筛查项目需要高通量检测时尤为突出；3）常用的液态保存液（PreservCyt/TCT）以酒精为主要介质，因而受到常规过空运的选址，且在家庭使用时存在易燃风险。

对于自取样样本，我们将采用经检测和验证的样本劈分法，先将样本涂抹在 POI 卡上，然后再将附带剩余样本的取样刷放入 TCT 液中[43,44]。

为了将来的研究，我们还将在筛查对象完成自取样之后，安排医生在进行医生取样操作前，于放置阴道窥器前获得 2 份“模拟自取样”样本。这 2 份样本将作为“干刷样本”被置于塑料袋封存，分别于取样后 7 日内和 6 周以后检测[49]。

如上所述，由于大部分检测阳性的女性都只是非致癌性 HPV 感染，且可以自发清除，因此 HPV 检测作为初筛的主要问题是特异性问题。为了解决这个问题，我们曾经研究过用 HPV mRNA 检测作为初筛[46]。此外，我们还对 Cervista 和 MALDI-TOF 初筛后用 HPV16/18 型进行二次分流进行过研究[47,48]。本次研究将使我们以往在自取样、Cobas（分别报告 HPV16 和 18 型）、 Aptima GT（APTIMA HR 检测的一项二次检测，可分别检测 HPV16 和 HPV18/45（18/45 一起检测）），及 SeqHPV 检测（可分别测序检测 14 种高危亚型 HPV）方面所做的研究工作得到进一步扩展。

3. 研究设计

3.1 方法

这是一项多中心、跨平台交叉比对的人群宫颈癌筛查研究。该研究将为过去 3 年内没有进行过宫颈癌筛查的 10,000 名女性进行筛查。该研究项目将提交中国北京大学深圳医院和美国克利夫兰医学中心伦理委员会（IRB）审批，并将已经过了 WHO 认定的 Clinical Clinic Trial Rigistry 网站上登记注册。

该临床研究将包括面向所有筛查女性的 1 次阴道自取样本和 1 次医生取样，以及对一个高危险地区筛查女性的 2 次模拟自取样。所有样本都将进行进行两种 HPV 检测。所有液体自取样样本、医生取样样本和模拟自取样样本的任一 HPV 检测结果阳性的筛查女性都将被回叫进行阴道镜检查和活检。

1) 募集 10,000 名女性参加本研究筛查（见附件筛查流程图）

   - 入选条件：年龄 30-59 周岁，至少 3 年内没有参加过筛查，无全子宫切除手术史，无盆腔放射治疗史，未处在孕期，且同意在初筛结果显示需要时接受本项目安排的阳性者临床处理的女性。
   - 排除条件：年龄小于 30 周岁大于 59 周岁，过去 3 年内曾参加过宫颈癌筛查，有过子宫全切手术史，有过盆腔放射治疗史，或不同意在初筛结果显示需要时接受本项目安排的阳性患者处理的女性。

2) 签知情同意书

3) 个人信息登记

4) 参加筛查女性采集 1 份阴道自取样本，首先涂布于 POI 卡，然后将刷子在 TCT 液体样本瓶内充分搅动（劈分样本）。涂卡样本采用已经验证过的“Just for Me”™自取样套装，里面含有一枝取样刷、一张 POI 卡和一个信封，所有内容都已贴好电脑条形码。套装外包装上印有图文版《自取样说明书》。以 PreseavCyt 为保存液的样本也将使用 POI 刷获取，样本将直接放置于 6mlPreservCyt 样本瓶。

- *#1 自取样本*：首先将取样刷上样本涂抹在 POI 卡上，然后将取样刷在装有 6ml PreservCyt/TCT 转换液的小瓶中搅动（劈分样本）。

  自取样 POI 卡样本和 TCT 保存液样本将采用 a) Cobas4800 和 b) SeqHPV 检测进行检测。

5) *i* 自取样完成后，所有现场的医生将为筛查女性放置阴道窥器，然后自每位筛查女性获取一份宫颈样本，放入 20ml ThinPrep® PreservCyt® (TCT) （HOLOGIC）小瓶中备检，只有内蒙古现场，医生将在放置阴道窥器之前首先获取 2 份“模拟阴道自取样”样本，待样本自然风干后将取样刷置于塑料单封存，分别用于在取样后 7 天之内和 6 周之后进行 a）Cobas4800 和 b）SeqHPV 的检测。

   医生取样样本将进行 a) Cobas4800 和 b) SeqHPV 检测。

   采用 Hologic 公司的 I2 Imager（计算机辅助细胞学检测）的细胞学检测将被用于以后的研究，而非本研究中阳性患者处理的参考。细胞学检查的主要目的是探索非 16/18 型 HPV 阳性患者的二次分流方案。

   POI 卡处理程序：每张卡将用手动打孔取 4 个样本片，放到 96 孔板的一个孔内，然后注入 100 微升灭菌水洗浴一次；用消毒过的移液器和适配容量的枪头小心地将液体移除。DNA 洗提的过程为：在每个微孔中各加入 50 微升的灭菌水，将 96 孔板置于温浴箱内，首先以 56℃ 加热 30 分钟，再立刻以 95℃ 加热 15 分钟。将包括洗提到的 DNA 和样本片的 96 孔板置于离心机以 4000 转/分离心 30 分钟，之后将包含 DNA 的液体转移到另一个 96 孔板。任何需要保存的样本都将被置于-80℃ 冰箱保存备检。当抽样检测样本选定之后，96 孔板上相应样本编号的 DNA 液将被抽取 5ul 用于 SeqHPV 的 PRC 扩增，10ul 用于 Cobas 进行的“巢式”抽样检测。(需要提请注意的是，这个 DNA 加载量是 SeqHPV 检测的标准量，这一样本量已被初步证明和验证对 99%以上的样本都可获得满意的检测结果。我们前期的预实验也证明，自 POI 卡洗提到的 10ul 的 DNA 样本量对于 Cobas 也非常理想)。

检测流程图 (2 项巢式检测):

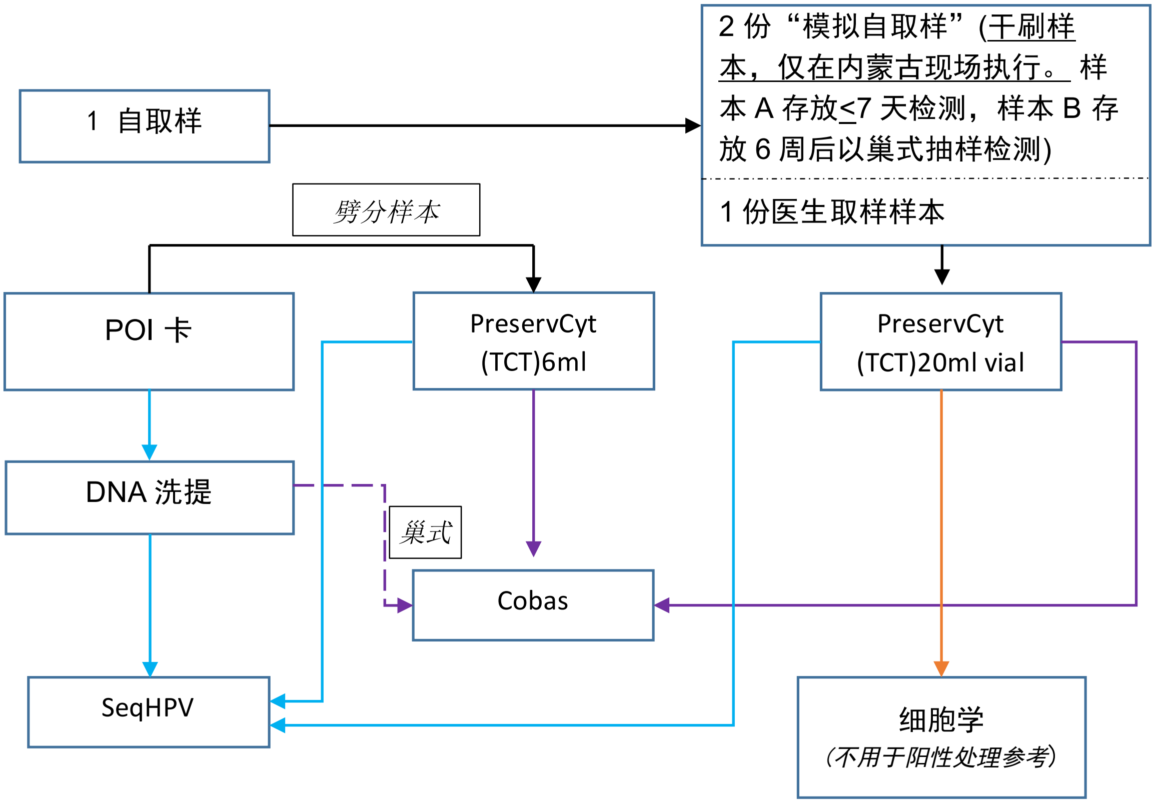

样本保存流程

1 份自取样本 -------涂抹在 ❶POI Card------- 残留的样本放入❷ 6 ml PreservCyt 转换液中；

2 份模拟阴道自取样样本（仅限于内蒙古现场）的干刷❸放入塑料袋封存备检：样本存放少于 7 天检测；样本 B 存放超过 6 周后采用巢式抽样检测；

1 份医生获取的宫颈样本------放入 ❹PreservCyt 20ml 中；

样本检测流程:

自取样本 1❶POI Card ------------------SeqHPV 全部 检测和 Cobas 巢式抽样检测；

自取样本 1❷PreservCyt 保存液(6ml) --- Cobas(1ml)和 SeqHPV 检测(1ml)检测；

干刷样本 A❸经保存液在处理后---------Cobas（1ml）和 SeqHPV（1ml）检测

干刷样本 B❸经保存液在处理后----巢式 Cobas（1ml）和 SeqHPV（1ml）检测

医生取样样本❹PreservCyt (20ml)---Cobas(1ml)、SeqHPV(1ml) 检测和细胞学检查

6) ❶-❹任一 hrHPV 检查阳性者都将被回叫行阴道镜检查和采用包括定点或随机活检+宫颈管诊刮术（ECC）在内的 POI 活检方案[49]进行的活检诊断。

7) 宫颈病变的处理将依据宫颈活检病理检查结果，参照北京大学深圳医院现行诊疗流程进行。

3.2 研究人群

本研究所涉及的10,000名女性将从中国深圳及5个筛查现场募集。这5个筛查现场是：1）北京市、2）上海市、3）河北省石家庄市、4）湖北省武汉市、5）内蒙古自治区鄂尔多斯市乌审旗。

筛查现场应能够代表：1）中国南北方发达、发展中和不发达地区；2）包括新近城市化的和依然从事农业生产的农村人口的地区；3）医疗资源相对充足、不足和匮乏的地区。

选择上海地区是因为：1）上海是中国最发达城市之一，代表中国东南方经济发达地区；2）有大量近年来城市化农村人口；3）其人均医疗资源在中国处于最高水平中。

选择北京地区是因为：1）北京是中国最发达城市之一，代表中国北方经济发达地区；2）有大量近年来城市化农村人口和农村人口；3）其人均医疗资源在中国处于最高水平中。

选择湖北省省会城市武汉是因为：1）武汉是中国中部典型的发展中城市，代表中国长江流域的中国大部分人口；2）周边有大量农村/农业人口；3) 是医疗资源相对不足的二线城市之一。

选择河北省省会城市石家庄的原因是：1）石家庄是典型的中国北方发展中城市；2）周边有大量农村/农业人口；3) 医疗资源相对不足的二线城市之一。

选责内蒙古自治区乌审镇的原因是：1）乌审镇是中国西北不发达城市之一，有大量农业人口；2）抽样调查 HPV 感染率高达 22%；3）大部分居民为农牧民；4）医疗资源严重不足（非常贫穷）的中国四线城市之一。

3.3 数据管理及数据统计方法

数据管理

我们将采用严格的数据管理系统。所有进入研究参加筛查的女性都分配有一个研究编码（ID），只有项目负责人（PIs）和筛查点负责人（The Site Coordinator）可以进入数据库，核校个人信息和研究编码。实验室只能通过研究编码识别样本和结果，不能接触个人信息。除了为病人提供医疗和随访的必需外，与筛查对象姓名和个人识别信息相关联的实验室信息均不会对外发布。所有与研究成果有关的报告、出版物和科学演讲搞都不会包括任何个人信息，因此筛查对象的个人识别信息不会被发布出去。

本研究的所有样本都将被跟踪。相关问卷调查和实验室数据将被填写到筛查现场的数据表中。这些数据表将被复制为一式两份，原件将移交给位于北京大学深圳医院的本项目数据管理

中心，以“同步双录入”方式录入“研究数据管理系统”。这些文件，所有的表格由研究管理人员锁存。

数据管理包括严格的质量控制程序，这些程序包括对筛查项目范围核对、完成情况评估和各选项间的吻合度的交叉检查。研究项目组的流行病学家和生物统计学家将对每个筛查点的数据管理员进行监督。所有的数据输入一个准确无误的数据库，并进行封存，作为项目统计分析的最终数据库。

数据分析

数据分析美国俄亥俄州克利夫兰市的隶属于美国克利夫兰医学中心妇女健康研究所的国际防癌组织和北京大学深圳医院协作完成。

统计学方法

本研究的主要结点是各项检测对于诊查宫颈高度病变（CIN3）或宫颈癌（CIN3+）的敏感性和特异性。本研究的次要结点将是各项高危 HPV 检测试验对诊查 CIN2+的敏感性和特异性。

我们对各项检测在本研究可能检出的 CIN3+的敏感性和特异性的差异的估计是基于人群筛查研究所获得的 hrHPV 感染发病率和 CIN3+（主要结点）发病率或 CIN2+发病率（次要结点）资料做出的。由于有 SHENCCAST II 数据[17]的引导，我们对 HPV 感染和 CIN3+发病率的估计可能会比很多其他研究更为准确。在 SHECCAST II 研究中[17]，我们筛查了人口特性与本研究的 4 个现场相近似的人群。而我们已知，本研究的第五个现场（内蒙古）的 HPV 感染率和癌前病变及浸润性癌变的发病率都明显高于 SHENCCAST II 筛查对象。内蒙古现场的选择将会弥补 SHENCCAST II 和本研究的筛查人口年龄差异所造成的发病率差异。

如同 SHENCCAST II 研究一样，我们将计算每项 hrHPV 检测的敏感性和特异性，并将应用逻辑回归分析为可能与每项检测方法的检测结果相关的因素做出相对危险度和 95%CI。

对比各项检测的敏感性和特异性所需要的样本规模的计算是根据目标人口的 hrHPV 感染率和研究结点病变（CIN3+或 CIN2+）发病率资料决定的。在 SHECCAST II 研究中，自取样阴道样本或医生取样宫颈口样本的 MALDI-TOF 或 Cervista HPV 检测所得到的 hrHPV 发病率均在 11.1%到 14.8%。SHENCCAST II 主要研究结点 CIN3+的发病率为 1.6%（141/8,556）；其次要结点 CIN2+的发病率为 2.7%（227/8,556）。SHENCCAST II 的研究结果呈示在表 1 中。本项目计划采用的 SeqHPV 检测是采用 SHENCCAST II 的样本开发和验证的，检测结果与 MALDI-TOF 相同[19]。

表 1: Cervicsta 和 MALDI-TOF HPV 检测的
自取样阴道样本和医生取样宫颈样本检测
对于诊查 CIN3+病变的命案性和特异性对照
(95% 置信区间与实际患者数量显示在括号中)

自取样阴道样本的 MALDI-TOF 检测和医生取样 Cervista 检测的
敏感性比较 McNemars p-值为 1.0，特异性比较的 McNemars p-值=<0.0001

**样本类别/HR-HPV 检测方法**	**≥CIN 3 病变敏感性 (%) (C.I.)(n)**	**≥CIN 3 病变特异性 (%) (C.I.)(n)**
自取样阴道样本/ Cervista	**70.9%** (62.7-78.3) (100/141)	**86.1%** (85.3-86.8) (7248/8415)
宫颈样本/ Cervista	**95.0%** (90.0-98.0) (134/141)	**90.3%** (89.6-90.9) (7598/8415)
自取样阴道样本/ MALDI-TOF	**94.3%** (89.1-97.5) (133/141)	**87.5%** (86.8-88.2) (7370/8415)
宫颈样本/ MALDI-TOF	**94.3%** (89.1-97.5) (133/141)	**89.4%** (88.7-90.0) (7526/8415)

上表各项数据的 hrHPV 敏感性的 95% CI 的跨度大约为 8%。这提示，对于一想涉及 10000 名女性，hrHPV 发病率为 11.1%-14.8%，CIN3+发病率为 1.6%的大型临床试验研究而言，敏感性相差 8%左右是可信的。表 1 中 hrHPV 特异性的 95%CI 跨度为 1.3%。鉴于该研究的每个受检者都有两份不同取样样本，诊查 CIN3+的特异性有可能相差接近 7%（如 94.3%（133/141）对 87.2%（123/141）而非 8%。

***首要研究目的：**评价 Cobas 4800 检测技术和 SeqHPV 检测技术检测自人群筛查中获取的阴道自取样本中检出宫颈高度病变（癌前病变）的效果。*

为评价各 HPV 检测平台的相对敏感性和特异性，我们将以宫颈活检病理结果为金标准，计算不同 HPV 检测平台的阳性率（敏感性）和假阳性率（特异性）。

***次级目标 1**: 评价不同检测方案对固体（滤纸卡）、干刷和液体样本保存介质在收集阴道自取样本和宫颈口医生取样样本时的差异。*

为确定对某一特定检测平台而言哪种样本介质更适合采集和储存自取样本。我们将比较不同样本介质在诊断 CIN2+和 CIN3+病变的相对敏感性以及在 HPV 检测中样本量不足的例数。

***次级目标 2:** 评价以 Cobas 和 SeqHPV 三种 HPV 检测方法初筛后，以细胞学和 HPV 亚型作为二次筛查的作用。*

本研究目标的重点在于探索一种可行方案，在尽量减小筛查敏感性损失的同时获得最高特异性。McNeemar’s 检验和卡方检验将被分别用于成对检测结果和独立分组检测结果对≥CIN 3 病变的敏感性和特异性的比较。细胞学检测结果将被用于非 16/18 型 HPV 阳性患者的分流管理。HPV 感染型别的分析将参照 SeqHPV 检测报告的 14 个型别；16、18 型的确定将参照 SeqHPV 和 Cobas 的检测报告。

我们将采用微观成本分析技术和决策分析技术对"筛查体统"进行成本效益分析。我们将对不同初筛方案、不同二次筛查方案、以及整个筛查系统的花费和成本效益进行评估。 成本的测算将采用微观成本方法进行。贯穿整个筛查过程的所有医疗花费都将得到跟踪，用于获得一个"人均筛查费用"，以便与平均每例 10 美元的政府宫颈癌筛查财政投入进行比较。需要进行分析的花费包括：（1）直接医疗费用（例如：取样物品套装、样本运输、样本分流、治疗选择、医护人员时间、医用材料及设备等)；（2）非直接医疗费用（例如：病人交通等)；（3）病人时间花费（例如：就诊交通、候诊和接受筛查时间)；（4）筛查项目执行成本（例如管理层次产生的费用，而不是某个医疗操作相关的费用，例如：培训、教育、宣传等)。为了评价筛查系统的成本，我们将把主要反映项目实际支出的财务成本自包含项目执行各方贡献和共享的资源价值的经济成本中分离出来，以更充分地评价机会成本。我们将测算随时间推移的预算影响和经济成本的影响，这些测算将从不同假设筛查方案下的筛查参与度、随访依从性、减轻疾病负担的效果三个层面进行。

4. 受检者安全保护

本研究是一项非进入性实验研究，仅自受检者获取宫颈或阴道样本。AE/SAE 与此类诊断性检测无关。因此，本研究中预期不会发生与本研究计划试验的诊断性检测有关的任何受检者直接伤害。

5. 符合性声明

美国克利夫兰医学中心（Cleveland Clinic）和北进大学深圳医院的伦理委员会（IRB）对本研究的批准完全满足以下符合性声明中所涵盖的所有要求：

本项研究将完全按照本研究方案、以及最新版本的赫尔辛基宣言，ICH GCP、和相关的地方法律法规执行。

项目文件的提交：研究开始前，本研究的研究方案、受检者个人信息登记表和相关知情同意书、以及法律法规要求的一切与研究有关的研究文件都将提交伦理委员会和相关政府权力机构，并获得书面审批。任何需要伦理委员会重新考量的研究方案的修改、更新和补充也将在实施之前提交伦理委员会获得书面审批。此外，研究小组还将向相关权力机构定期提交研究报告（中期报告或结题报告)。

受检者告知和知情同意：研究小组将向受检者或其合法代表人口头和书面告知本研究的目标、研究流程、以及因研究执行控制的原因本研究资料或需在保证受检者个人资料保密的前提下向第三方提供的可能性。

在任何研究活动开始前，受检者或其合法代表人需要签署书面知情同意书。参与本研究项目完全出于个人自愿。本研究受检者有权在在任何时候撤销其参与本研究的意愿，且不会对其个人未来的医疗保健产生任何影响。

临床试验结果的发布：本研究的数据将记载于临床试验报告，如果可能，也将会通过学术论文（在学术期刊或学术会议上）发表。

参考文献

1. Cuzick J, Clavel C, Petry KU, Meijer CJ, Hoyer H, Ratnam S, Szarewski A, Birembaut P, Kulasingam S, Sasieni P, Iftner T. Overview of the European and North American studies on HPV testing in primary cervical cancer screening. Int J Cancer. 2006;119(5):1095-101.
2. Mayrand MH D-FE, Rodrigues I,Walter SD, Hanley J, Ferenczy A, Ratnam S,Coutlee F,Franco EL; cervical cancer screening trial study group. Human Papillomavirus DNA versus Papanicolaou screening tests for cervical cancer. N Engl J Med. 2007;357(16):1579-88.
3. Naucler P, Ryd W, Tornberg S, Strand A, Wadell G, Elfgren K, Radberg T, Strander B, Johansson B, Forslund O, Hansson BG, Rylander E, Dillner J. Human papillomavirus and Papanicolaou tests to screen for cervical cancer. N Engl J Med. 2007;357(16):1589-97.
4. Ronco G, Giorgi-Rossi P, Carozzi F, Confortini M, Dalla Palma P, Del Mistro A, Ghiringhello B, Girlando S, Gillio-Tos A, De Marco L, Naldoni C, Pierotti P, Rizzolo R, Schincaglia P, Zorzi M, Zappa M, Segnan N, Cuzick J, New Technologies for Cervical Cancer screening Working G. Efficacy of human papillomavirus testing for the detection of invasive cervical cancers and cervical intraepithelial neoplasia: a randomised controlled trial. Lancet Oncol. 2010;11(3):249-57.
5. Rijkaart DC, Berkhof J, Rozendaal L, van Kemenade FJ, Bulkmans NW, Heideman DA, Kenter GG, Cuzick J, Snijders PJ, Meijer CJ. Human papillomavirus testing for the detection of high-grade cervical intraepithelial neoplasia and cancer: final results of the POBASCAM randomised controlled trial. Lancet Oncol. 2012;13(1):78-88.
6. Ronco G, Dillner J, Elfstrom KM, Tunesi S, Snijders PJ, Arbyn M, Kitchener H, Segnan N, Gilham C, Giorgi-Rossi P, Berkhof J, Peto J, Meijer CJ, the International HPVswg. Efficacy of HPV-based screening for prevention of invasive cervical cancer: follow-up of four European randomised controlled trials. Lancet. 2013.
7. Sankaranarayanan R, Nene BM, Shastri SS, Jayant K, Muwonge R, Budukh AM, Hingmire S, Malvi SG, Thorat R, Kothari A, Chinoy R, Kelkar R, Kane S, Desai S, Keskar VR, Rajeshwarkar R, Panse N, Dinshaw KA. HPV screening for cervical cancer in rural India. N Engl J Med. 2009;360(14):1385-94.
8. Castle PE, Wheeler CM, Solomon D, Schiffman M, Peyton CL, Group A. Interlaboratory reliability of Hybrid Capture 2. Am J Clin Path. 2004;122(2):238-45.
9. Carozzi FM, Del Mistro A, Confortini M, Sani C, Puliti D, Trevisan R, De Marco L, Tos AG, Girlando S, Palma PD, Pellegrini A, Schiboni ML, Crucitti P, Pierotti P, Vignato A, Ronco G. Reproducibility of HPV DNA Testing by Hybrid Capture 2 in a Screening Setting. Am J Clin Path. 2005;124(5):716-21.
10. Dillner J, Rebolj M, Birembaut P, Petry KU, Szarewski A, Munk C, de Sanjose S, Naucler P, Lloveras B, Kjaer S, Cuzick J, van Ballegooijen M, Clavel C, Iftner T, Joint European Cohort S. Long term predictive values of cytology and human papillomavirus testing in cervical cancer screening: joint European cohort study. BMJ. 2008;337:a1754.

11. Andrae B, Kemetli L, Sparen P, Silfverdal L, Strander B, Ryd W, Dillner J, Tornberg S. Screening-preventable cervical cancer risks: evidence from a nationwide audit in Sweden. J Natl Cancer Inst. 2008;100(9):622-9.
12. Shi JF, Belinson JL, Zhao FH, Pretorius RG, Li J, Ma JF, Chen F, Xiang W, Pan QJ, Zhang X, Zhang WH, Qiao YL, Smith JS. Human papillomavirus testing for cervical cancer screening: results from a 6-year prospective study in rural China. Am J of Epidemiol. 2009;170(6):708-16.
13. Saslow D, Solomon D, Lawson HW, Killackey M, Kulasingam SL, Cain J, Garcia FA, Moriarty AT, Waxman AG, Wilbur DC, Wentzensen N, Downs LS, Jr., Spitzer M, Moscicki AB, Franco EL, Stoler MH, Schiffman M, Castle PE, Myers ER, Committee A-A-ACCG. American Cancer Society, American Society for Colposcopy and Cervical Pathology, and American Society for Clinical Pathology screening guidelines for the prevention and early detection of cervical cancer. CA: a cancer journal for clinicians. 2012;62(3):147-72.
14. Kim JJ, Brisson M, Edmunds WJ, Goldie SJ. Modeling cervical cancer prevention in developed countries. Vaccine. 2008 Aug 19;26 Suppl 10:K76-86. doi: 10.1016/j.vaccine.2008.06.009.
15. Zhao FH, Lewkowitz AK, Chen F, Lin MJ, Hu SY, Zhang X, Pan QJ, Ma JF, Niyazi M, Li CQ, Li SM, Smith JS, Belinson JL, Qiao YL, Castle PE. Pooled analysis of a self-sampling HPV DNA Test as a cervical cancer primary screening method. J Natl Cancer Inst. 2012;104(3):178-88.
16. Belinson JL, Du H, Yang B, et al. Improved sensitivity of vaginal self-collection and high-risk human papillomavirus testing. Int J Cancer 2012; 130:1855-1860.
17. Du H, Yi J, Wu R, et al. A New PCR based Mass Spectrometry System for High-Risk HPV Part II – Clinical Trial. Am J Clin Pathol.2011;136:920-923
18. Belinson JL, Hu S, Niyazi M, et al. Prevalence of type-specific human papillomavirus in endocervical, upper and lower vaginal, perineal, and vaginal self-collected specimens; implications for vaginal self-collection. Int J Gyn Cancer 2010 Sept 1;127:1151-1157.
19. Yi X, Zou J,Xu J, et al. Development and validation of a new HPV genotyping assay based on next generation sequencing. Am J Clin Pathol. 2014;141:796-804.
20. Wright TC, Stoler MH, Behrens CM, Sharma A, Zhang G, Wright TL. Primary cervical cancer screening with human papillomavirus: end of study results from the ATHENA study using HPV as the first-line screening test. Gynecol Oncol. 2015 Feb;136(2):189-97. doi: 10.1016/j.ygyno.2014.11.076. Epub 2015 Jan 8.
21. Castle PE, Stoler MH, Wright TC Jr, Sharma A, Wright TL, Behrens CM. Performance of carcinogenic human papillomavirus (HPV) testing and HPV16 or HPV18 genotyping for cervical cancer screening of women aged 25 years and older: a subanalysis of the ATHENA study. Lancet Oncol. 2011 Sep;12(9):880-90. doi: 10.1016/S1470-2045(11)70188-7. Epub 2011 Aug 22.
22. Arbyn M, Verdoodt F, Snijders PJ, Verhoef VM, Suonio E, Dillner L, Minozzi S, Bellisario C, Banzi R, Zhao FH, Hillemanns P, Anttila A. Accuracy of human papillomavirus testing on self-collected versus clinician-collected samples: a meta-analysis. Lancet Oncol. 2014 Feb;15(2):172-83. doi: 10.1016/S1470-2045(13)70570-9. Epub 2014 Jan 14.

23. Kailash U, Hedau S, Gopalkrishna V, Katiyar S, Das BC. A simple 'paper smear' method for dry collection, transport and storage of cervical cytological specimens for rapid screening of HPV infection by PCR. J Med Microbiol. 2002 Jul;51(7):606-10.
24. Gustavsson I, Lindell MF, Wilander EF, Strand AF, Gyllensten U. Use of IFTA card for dry collection, transportation and storage of cervical cell specimen to detect high-risk HPV. J Clin Virol. 2009: 46: 112-6.
25. Gustavsson I, Sanner KF, Lindell MF, Strand AF, Olovsson MF, Wikstrom IF, et al. Type-specific detection of high-risk human papillomavirus (HPV) in self-sampled cervicovaginal cells applied to iFTA elute cartridge. J Clin Virol 2011; 51:255-8.
26. Lenselink CH, de Bie RP, van Hamont D, Bakkers JM, Quint WG, Massuger LF, Bekkers RL, Melchers WJ: Detection and genotyping of human papillomavirus in self-obtained cervicovaginal samples by using the FTA cartridge: new possibilities for cervical cancer screening. J Clin Microbiol 2009, 47:2564–70.
27. De Bie, R.P., Schmeink CE, Bakker JM, Snijder PJ, Quint WG, Massuger, L.F., Bekkers, R.L., Melchers, W.J., 2011. The indicating FTA elute cartridge a solid sample carrier to detect high-risk HPV and high-grade cervical lesions. J. Mol. Diagn. 2011, 13: 371–6.
28. Gonzalez P, Cortes B, Quint W, Kreimer AR, Porras C, Rodriguez AC, et al. Evaluation of the IFTA carrier device for human papillomavirus testing in developing countries. J Clin Microbiol. 2012 Dec; 50(12):3 870-6.
29. Geraets DT, van Baars R, Alonso I, Ordi J, Torne A, Melchers WJ, et al. Clinical evaluation of high-risk HPV detection on self-samples using the indicating FTA-elute solid-carrier cartridge. J Clin Virol. 2013 Jun; 57(2):125-9.
30. Guan Y, Gravitt PE, Howard R, Eby YJ, Wang S, Li B, et al. Agreement for HPV genotyping detection between self-collected specimens on a iFTA cartridge and clinician-collected specimens. J Virol Methods. 2013 Apr; 189(1):167-71.
31. Wang SM, Hu SY, Chen F, Chen W, Zhao FH, Zhang YQ, et al. Clinical evaluation of human papillomavirus detection by careHPV test on physician-samples and self-samples using the indicating FTA elute card. Asian Pac J Cancer Prev. 2014;15(17):7085-9.
32. Gyllensten U, Gustavsson I, Lindell M, Wilander E. Primary high-risk HPV screening for cervical cancer in post-menopausal women. Gynecol Oncol. 2012 May;125(2):343-5.
33. Phongsavan K, Gustavsson I, Marions L, Phengsavanh A, Wahlstrom R, Gyllensten U. Detection of human papillomavirus among women in laos: Feasibility of using filter paper card and prevalence of high-risk types. Int J Gynecol Cancer. 2012 Oct;22(8):1398-406.
34. Santos CR, Franciscatto LG, Barcellos RB, Almeida SE, Rossetti ML. Use of FTA elute card impregnated with cervicovaginal sample directly into the amplification reaction increases the detection of human papillomavirus DNA. Braz J Microbiol. 2012 Jan;43(1):389-92.
35. Mei JV, Alexander JR, Adam BW, Hannon WH. Use of filter paper for the collection and analysis of human whole blood specimens. J Nutr. 2001 May;131(5):1631S-6S.
36. Beebe JL, Briggs LC. Evaluation of enzyme-linked immunoassay systems for detection of human immunodeficiency virus type 1 antibody from filter paper disks impregnated with whole blood. J Clin Microbiol. 1990 Apr;28(4):808-10.

37. Ayele W, Schuurman R, Messele T, Dorigo-Zetsma W, Mengistu Y, Goudsmit J, et al. Use of dried spots of whole blood, plasma, and mother's milk collected on filter paper for measurement of human immunodeficiency virus type 1 burden. J Clin Microbiol. 2007 Mar;45(3):891-6.
38. Fiscus SA, Cheng B, Crowe SM, Demeter L, Jennings C, Miller V, et al. HIV-1 viral load assays for resource-limited settings. PLoS Med. 2006;3(10):e417.
39. Pitcovski J, Shmueli E, Krispel S, Levi N. Storage of viruses on filter paper for genetic analysis. J Virol Methods. 1999;83(1-2):21-6.
40. Smit PW, Elliott I, Peeling RW, Mabey D, Newton PN. An overview of the clinical use of filter paper in the diagnosis of tropical diseases. Am J Trop Med Hyg. 2014 Feb;90(2):195-210.
41. Maurer K , Luo H, Shen Z, Wang G, Wang G, Du H, Qu X, Wu R, and Belinson J. The Development and Evaluation of a New Solid Media Specimen Transport Card for Population Based Cervical Cancer Prevention. (Manuscript in preparation as of 6-19-2015).
42. Hongxue Luo, Hui Du, Kathryn Maurer, Jerome L.Belinson, Guixiang Wang, Zhihong Liu, Lijie Zhang, Yanqiu Zhou, Chun Wang, Jinlong Tang, Xinfeng Qu, Ruifang Wu. An evaluation of the Cobas4800 HPV test on cervico-vaginal specimens in liquid versus solid transport media. Accepted Jan. 2016, Plos One.
43. Biscotti CV, O’Brien DL, Gero MA, Gramlich TL, Kennedy AW, Easley KA: Thin-layer Pap test vs. conventional Pap smear: Analysis of 400 split samples. J Reprod Med 2002;47:9-13.
44. Hutchinson ML, Agarwal P, Deanult T, Berger B, Cibas ES: A new look at cervical cytology: ThinPrep multicenter trial results. Acta Cytol 1992;36:499-504.
45. Rosa Catarino, Pierre Vassilakos, Aline Bilancioni, Mathieu Vanden Eynde, Ulrike Meyer-Hamme, Pierre-Alain Menoud, Frederic Guerry, Patrick Petignat. Self-sampling methods for human papillomavirus detection: dry swabs versus FTA cartridge.PLOS One; doi:10.1371/journal.pone.0143644. Dec.2,2015
46. Wu R, Belinson SE, Du H, et al. Human papillomavirus (HPV) mRNA assay for cervical cancer screening:The Shenzhen Cervical Cancer Screening Trial I (SHENCCAST I). Int J Gynecol Cancer. 2010;20:1411-1414.
47. Wu R, Du H, Belinson SE, et al. Secondary screening after primary self-sampling for human papillomavirus from SHENCCAST II. J Low Genit Tract Dis. 2012;16:416-420.
48. Goodrich SK, Pretorius RG, Du H, et al. Triage of women with negative cytology and positive high-risk HPV: An analysis of data from the SHENCCAST II/III studies. J Low Genit Tract Dis. 2014;18:122-127.
49. Belinson JL, Qiao YL, Pretorius RG, Zhang WH et al: Shanxi Province Cervical Cancer Screening Study. A Cross-Sectional Comparative Trial of Multiple Techniques to Detect Cervical Intraepithelial Neoplasia. Gynecol Oncol 83: 439-44, Nov 2001.
